# Influence of Maternal Breast Milk and Vaginal Microbiome on Neonatal Gut Microbiome: a Longitudinal Study during the First Year

**DOI:** 10.1128/spectrum.04967-22

**Published:** 2023-04-17

**Authors:** Chandrika Bhattacharyya, Diganta Barman, Devashish Tripathi, Soumita Dutta, Chandra Bhattacharya, Mahabub Alam, Parveena Choudhury, Utpala Devi, Jagadish Mahanta, Reeta Rasaily, Analabha Basu, Suman K. Paine

**Affiliations:** a National Institute of Biomedical Genomics, West Bengal, India; b Department of Paediatrics, Gauhati Medical College, Assam, India; c Regional Medical Research Centre, Indian Council of Medical Research, Assam, India; d Maternal and Child Health, Indian Council of Medical Research, New Delhi, India; Karolinska Institutet

**Keywords:** maternal microbiome, neonatal, neonatal stool, breast milk, gut microbiome

## Abstract

It is believed that establishment of the gut microbiome starts very early in life and is crucial for growth, immunity, and long-term metabolic health. In this longitudinal study, we recruited 25 mothers in their third trimester, of whom 15 had vaginal delivery while 10 had an unplanned cesarean section (C-section). The mother-neonate pairs were followed for 1 year, and we generated 16S metagenomic data to study the neonatal gut microbiome along with mother’s breast milk and vaginal microbiomes through 12 months after delivery, at 1, 3, 6, and 12 months. We inferred (i) mode of delivery is an important factor influencing both composition and entropy of the neonatal gut microbiome, and the genus Streptococcus plays an important role in the temporal differentiation. (ii) Microbial diversity monotonically increases with age, irrespective of the mode of delivery, and it is significantly altered once exclusive breastfeeding is stopped. (iii) We found little evidence in favor of the microflora of mother’s breast milk and a vaginal swab being directly reflected in the offspring’s gut microbiome; however, some distinction could be made in the gut microbiome of neonates whose mothers were classified as community state type III (CSTIII) and CSTIV, based on their vaginal microbiomes. (iv) A lot of the mature gut microbiome is possibly acquired from the environment, as the genera *Prevotella* and *Faecalibacterium*, two of the most abundant flora in the neonatal gut microbiome, are introduced after initiation of solidified food. The distinction between the gut microbiome of babies born by vaginal delivery and babies born by C-section becomes blurred after introduction of solid food, although the diversity in the gut microbiota drastically increases in both cases.

**IMPORTANCE** Gut microbiome architecture seems to have a potential impact on host metabolism, health, and nutrition. Early life gut microbiome development is considered a crucial phenomenon for neonatal health as well as adulthood metabolic complications. In this longitudinal study, we examined the association of neonatal gut microbiome entropy and its temporal variation. The study revealed that adult-like gut microbiome architecture starts taking shape after initiation of solidified food. Further, we also observed that the difference of microbial diversity was reduced between vaginally delivered and C-section babies compared to exclusive breastfeeding tenure. We found evidence in favor of the inheritance of the microflora of mother’s posterior vaginal wall to the offspring’s gut microbiome.

## INTRODUCTION

The diverse microbial ecosystem in the human body represents a symbiotic evolutionary process which facilitates host immunity, growth, and survival (1, 2). Through the different stages of life, the host microbiome plays an important role in nutrition, maintaining the homeostasis between the host’s metabolome and immunological components (3, 4). Gut microbial colonization is probably initiated during the gestational period *in utero*, and myriad factors are contributors to the neonatal microbial ecology (5). These factors include the following: (i) duration of gestation (term or preterm birth), (ii) mode of delivery (vaginal delivery [VD] or cesarean section [CS]), (iii) maternal nutrition, and (iv) immune status, as well as (v) microbiome of vaginal swab (VS) samples, particularly for the VD babies, and (vi) breast skin and breast milk (BM) of the mother, particularly for the breastfed babies.

To identify the factors influencing the neonatal gut microbiome (GM), we designed a prospective mother-neonate pair longitudinal study. We recruited 25 mothers who were in the third trimester of their pregnancies. Of these 25 mothers, 15 had VD while 10 had to go through unplanned CS.

There is lots of empirical evidence that exclusive breastfeeding for neonates’ results in improved cognitive and immunological development (6). In the long term, it protects the individual against a variety of respiratory and gastrointestinal infections, as well as metabolic diseases (7) of adulthood (8). Studies have quantified striking differences among the neonatal GM between formula-fed and exclusively BM-fed babies. If the BM microbiome impacts the GM of babies, then the reduced proportion of commensals in BM microbiome of mothers having CS (MoCS) compared to mothers having VD (MoVD) may also have some impact on neonatal development, despite their babies having similar exposure to breastfeeding.

We found neonatal GM to be sensitive to the mode of delivery of the offspring and tried to identify associated factors in the longitudinal dynamics of GM with maternal microflora, both BM and VS. As it is known that the BM of the mother changes its composition to match the age and various nutritional requirements of the child, we characterized the impact of the BM microbiome of mothers (both MoVD and MoCS) at different time points of the lactation phase, ideally after introduction of solid food and increased food diversity for the child. We also compared neonatal GM at year 1 (12 months) as it reflected possible changes in GM after initiation of solidified food.

**FIG 1:**
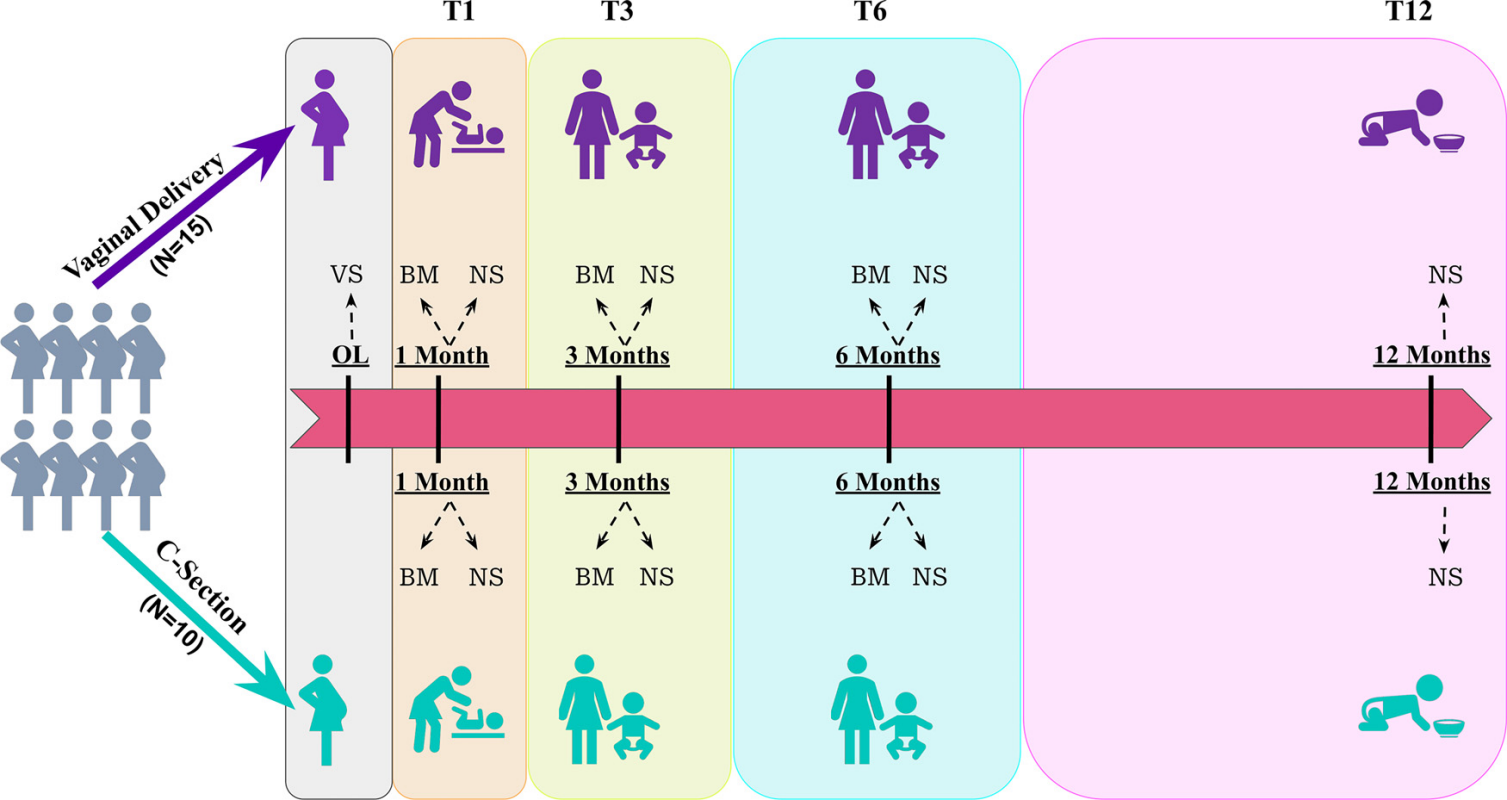
A longitudinal study on the neonatal GM in a mother-child paired design. Initially, 25 pregnant mothers were enrolled. Based on the mode of delivery, they were classified as vaginal delivery (VD) or C-section delivery (CS). A vaginal swab (VS) was collected at onset of labor (OL) for the mothers undergoing VD. All the mother-neonate pairs were followed and samples were collected: (1) breast milk (BM) from all mothers who had VD or CS, at 3 different time periods, T1, T2, and T3. (2) Neonatal stool (NS) from all children at 4 different time periods, T1, T2, T3, and T4.

We followed these mother-neonate pairs and studied the microbiome in (i) VS of mothers who had vaginal delivery (MoVD-VS); (ii) the microbiome in BM of mothers at 3 time points, <1 month after birth of the child (T1), ~3 months after birth of the child (T3), and ~6 months after birth of the child (T6), who had either vaginal delivery (MoVD-BM) or who had cesarean section (MoCS-BM); and (iii) the microbiome in neonatal stool (NS) of their children over 4 different time periods, at T1, T3, and T6 (as stated above) and at ~12 months after birth of the child (T12), who were born either via VD (nnVD-NS) or via CS (nnCS-NS). (A detailed flowchart of the designed study is available in Fig. 1; see also Table S1 in the supplemental material).

Infants start to acquire commensals, particularly *Lactobacillus*, during birth, and a likely possible source is the mothers’ vaginal microbiome (VM) (9). A previous report documented that the microbial architecture of the VM is broadly classified in five community state types (CST) that depend on the presence of *Lactobacillus* species (10). Hence, the maternal microbiome, particularly the VM and BM microbiome, may impact neonatal GM development in early life. Our study design allowed us to ask questions related to the heritable component of the neonatal microbiome, like (i) how does microbial diversity of a neonate compare with the VS microbiome or BM microbiome of the biological mother relative to other nursing women? (ii) What are the specific operational taxonomic units (OTUs) that are most determining and present throughout the initial formation of the microbial ecology of the neonatal gut? (iii) How much of the stable OTUs in the neonatal gut resemble the mother’s BM or VS microflora?

We believe that answers to our questions will elucidate a deeper understanding and characterization of the neonatal GM and will enable designing better and efficient probiotics with the combination of key microflora that are essential in the development of a healthy gut.

## RESULTS

### Neonatal stool diversity: mode of delivery and lactation period.

**FIG 2:**
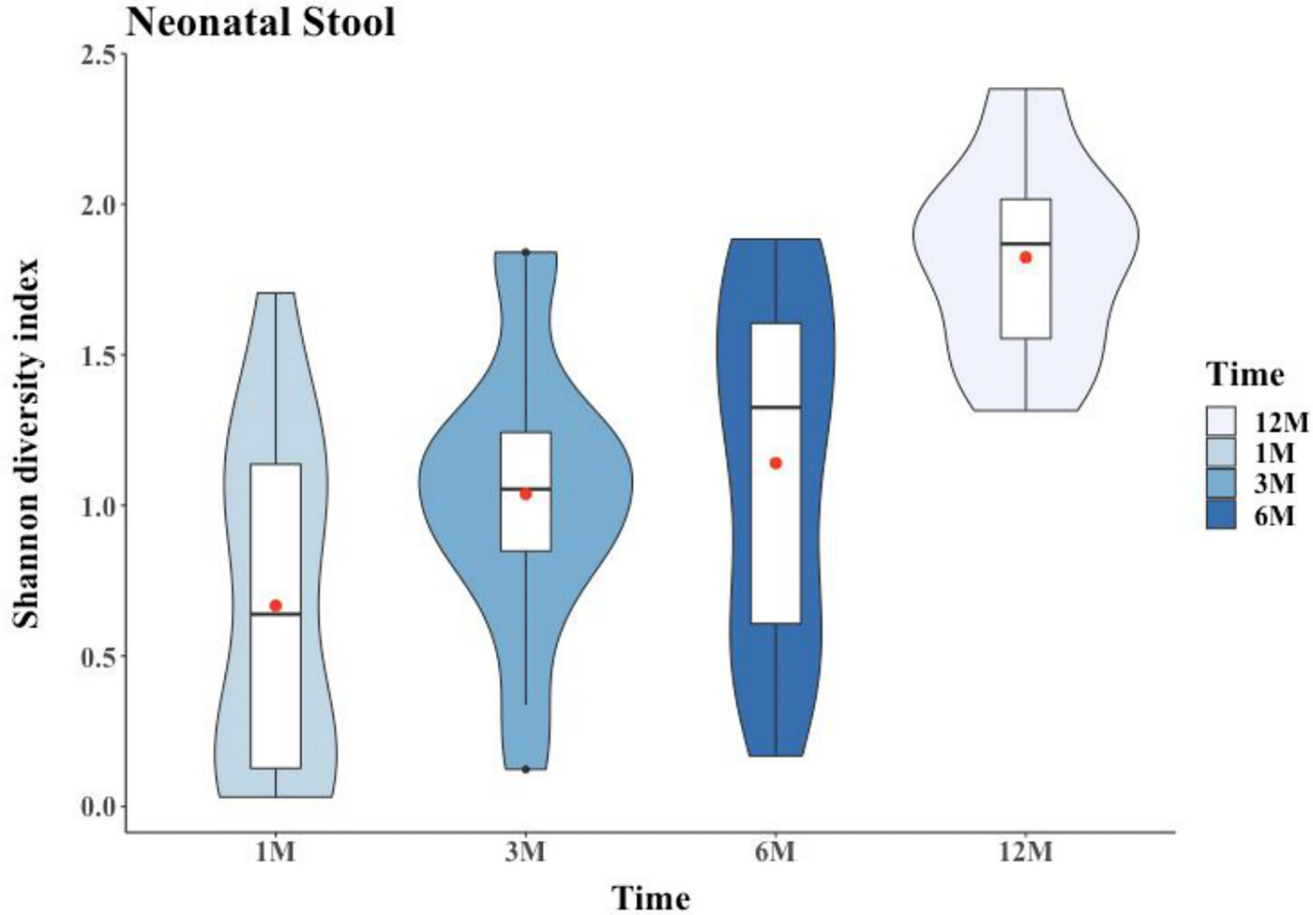
The dynamics of alpha-diversity (richness) of the neonatal gut microbiome with respect to different time points (1, 3, 6, and 12 months) was estimated through a Shannon diversity index (SDI) and illustrated by using violin plots. A Kruskal-Wallis test was employed for statistical comparisons. The diversity rose with time and peaks following the introduction of solidified food, i.e., at 12 months. The red dot represents the mean.

The overarching goal of this study was a coherent characterization of the neonatal GM based on exploring the diversity in NS. Considering the microbiome profile of the mother’s BM and VS and other cofactors like their mode of delivery (i.e., VD and CS), we documented the patterns and variability of microbial diversity in neonates over a span of three intervals for 1 year after birth (T1, T3, T6, and T12). A significantly lower Shannon’s diversity index (SDI) was observed among NS of neonates born through CS (nnCS-NS; mean ± standard deviation of 0.98 ± 0.51, range of 0.12 to 2.03) compared to NS of neonates born through VD (nnVD-NS; 1.29 ± 0.565, range of 0.03 to 2.38) during their first year of life (i.e., jointly for the four different time points; Kruskal-Wallis test; *P* = 0.03) (Fig. S2). The SDI was lowest at T1 (0.67 ± 0.56), and it significantly increased with time at T3 (1.04 ± 0.45) and T6 (1.14 ± 0.56); the SDI became highest, i.e., most stable, after initiation of solidified food at T12 (1.82 ± 0.32; Kruskal-Wallis test, *P* = 2 × 10^6^) (Fig. 2). This significant temporal increment of the SDI was also observed separately for both nnVD and nnCS babies (Kruskal-Wallis test; nnVD-NS, *P* = 3.5 × 10^−5^; nnCS-NS, *P* = 0.006027) (Fig. S3). However, the high SDI among nnCS gut flora was not always monotonous in the first three time intervals, but GM-SDI was elevated significantly after initiation of solidified food for both nnVD-NS and nnCS-NS babies. We did not find any significant difference in SDI between nnVD-NS and nnCS-NS at T1 and T12, but a significant rise was observed in SDI among nnVD neonates compared to nnCS, at least for T3 and T6, i.e., after birth during lactation of transition milk (Fig. S3).

**FIG 3:**
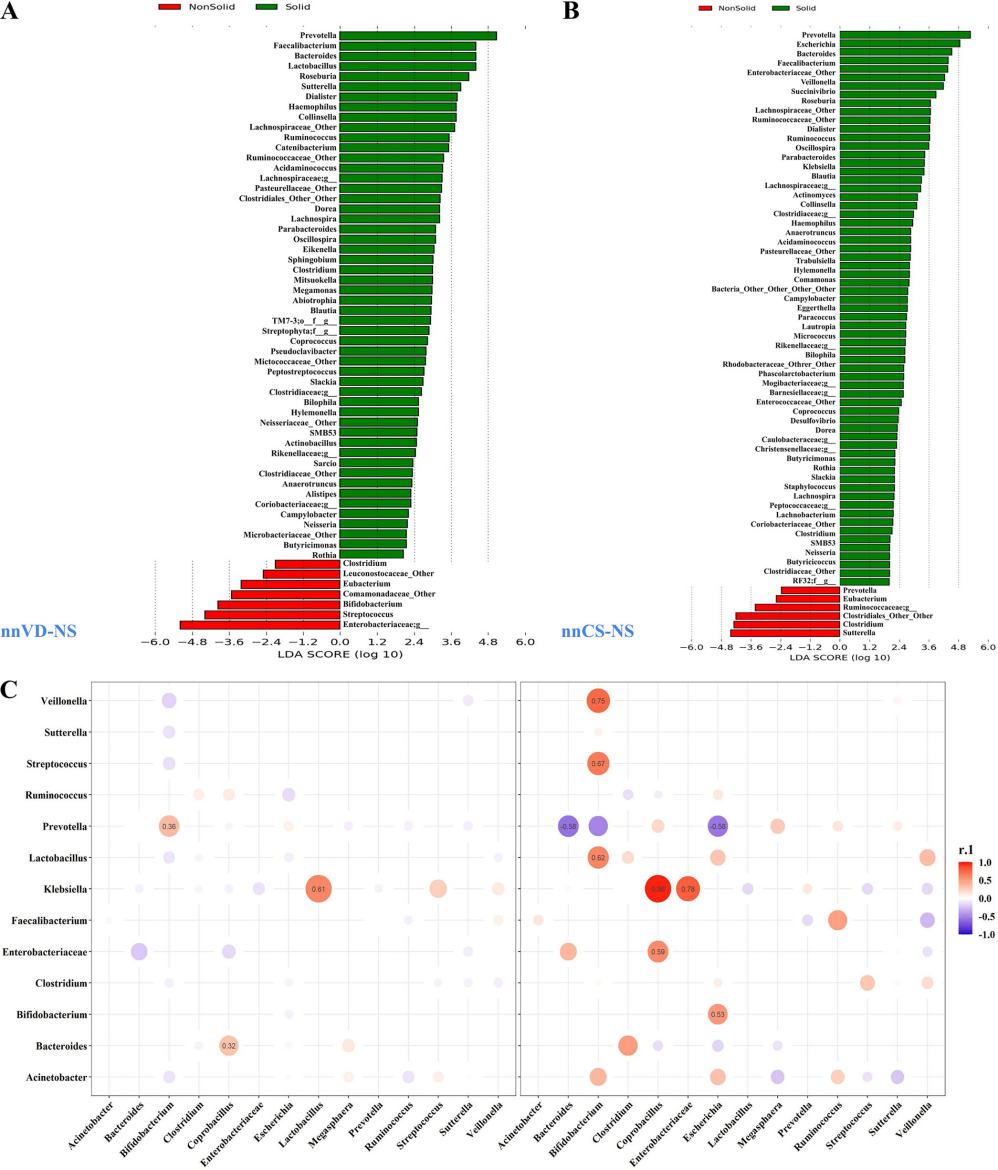
(A and B) Linear discriminant analysis (LDA) was used to identify the discriminative bacterial taxa between nonsolid (breast milk) and solid food among neonates delivered vaginally (nnVD-NS) (A) and via C-section (nnCS-NS) (B). The figure demonstrates that the bacteria taxon satisfied the default threshold of a 2.5-log LDA score. (C) Alteration of correlation among OTUs of neonatal gut microbiome compared between exclusively breastfed tenure (1 to 6 months) and after initiation of solidified food (12 months).

We characterized the species of bacteria that changed significantly after initiation of solid food. Depending on the diet of the neonate, we categorized nnNS data into two groups: (i) when they were exclusively on nonsolid food (T1, T3, and T6), and (ii) when solid food (T12) was introduced. Linear discriminant analysis (LDA) revealed that *Prevotella*, *Faecalibacterium*, *Bactereoidetes*, and *Roseburia* were present in significantly higher proportion in T12 compared to breastfeeding tenure among both the groups nnVD-NS and nnCS-NS (LDA score, >3.5 log_10_) (Fig. 3A and B). A striking difference was observed for the bacterial genus *Lactobacillus*, which exhibited an association among nnVD-NS after initiation of solidified food (LDA score, >3.5 log_10_), whereas its effect size was negligible (<1) among nnCS-NS. A significant reduction was observed for the genus *Bifidobacterium* (LDA score, >4.5), and Streptococcus and *Eubacterium* (LDA score, >3.5) among nnVD-NS after introduction of solidified food. Significant proportional reductions were found for the genera *Suterella*, *Clostridium* (LDA score, >3.5), *Eubacterium*, and *Prevotella* (LDA score, >2.5) among nnCS-NS upon initiation of solidified food compared to breastfeeding tenure (Fig. 3A and B). We have also observed a negative correlation for *Veillonella* and Streptococcus, with *Bifidobacterium* during breastfeeding tenure which shifted to a positive correlation after initiation of solidified food (12 months). In addition, a shift of positive to negative correlation was documented for the bacterial pairs of *Prevotella-Bifidobacterium* and Klebsiella-*Lactobacillus* between exclusively breastfeeding tenure groups (1 to 6 months) and after initiation of solidified food (12 months) (Fig. 3C). Detailed comparative notes at the genus level for all time points are provided in (Text S4 and Fig. S4).

**FIG 4:**
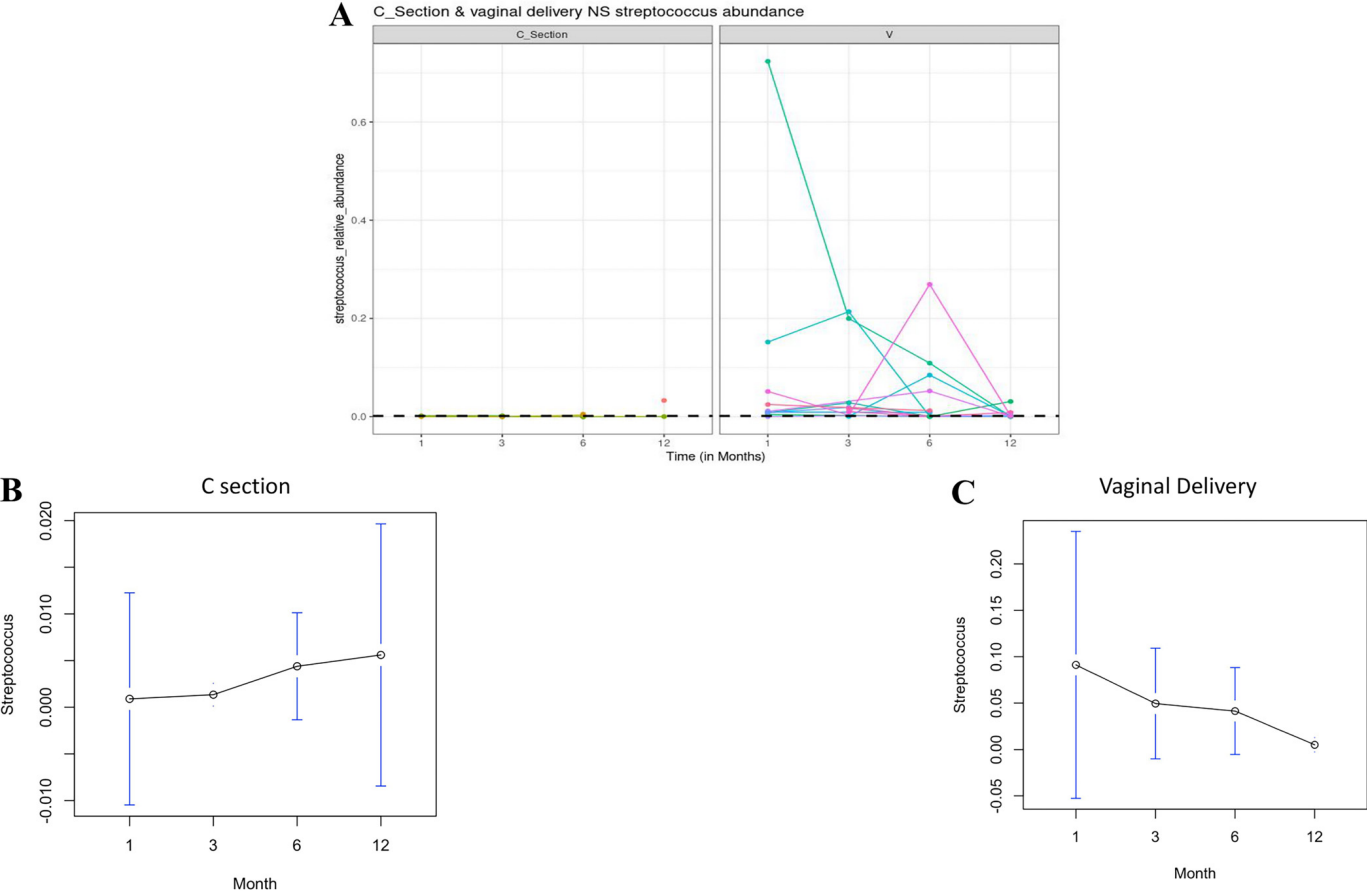
The microbiome interpretable temporal rule engine (MITRE) was used to identify Streptococcus as a key genus for microbiota time-series data linked to age of neonates among VD as well as CS infants. (A) The figure contains the individual trajectories of Streptococcus abundance in the two groups. The black dashed line represents an average abundance of 0.0013, which classifies the CS and VD groups. (B) Proportion of the genus Streptococcus in NS increased monotonously from month 1 to 12 among infants delivered by CS. For both panels A and B, the number of samples in each time points was as follows: *n* = 2 at 1 month, *n* = 4 at 3 months, *n* = 7 at 6 months, and *n* = 2 at 12 months. (C) The trend was antagonistic and the Streptococcus proportion decreased from month 1 to 12 among the VD group. The number of samples in each time points was as follows: *n* = 11 at 1 month, *n* = 10 at 3 months, *n* = 13 at 6 months, and *n* = 9 at 12 months.

To identify possible genera which temporally varied and differentiated between nnVD-NS and nnCS-NS, we employed a supervised machine-learning method, microbiome interpretable temporal rule engine (MITRE) (11). MITRE provided strong evidence against the possibility that the difference between nnVD-NS and nnCS-NS was only because of random fluctuations and identified the genus Streptococcus, with a Bayes factor of 0.0171, whose temporal difference (combining evidence from all time points) was the significant differentiator between nnVD-NS and nnCS-NS (Fig. 4A). The genus Streptococcus monotonically increased in proportion from T1 to T12 among the nnCS-NS group (Fig. 4B); however, it monotonically decreased in proportion during the same interval among the nnVD-NS group (Fig. 4C).

**FIG 5:**
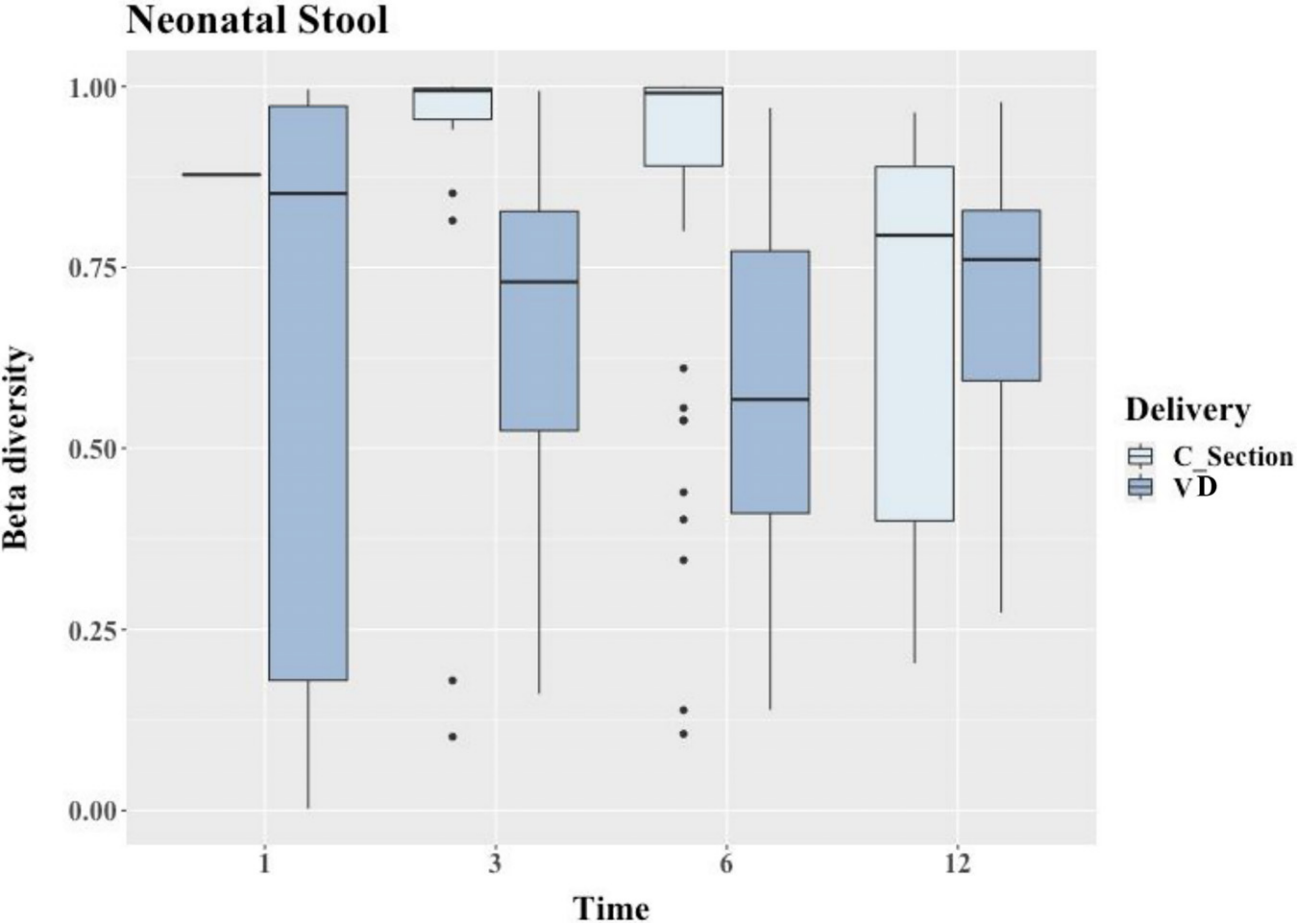
The interindividual variation of neonatal gut between vaginally delivered (VD) and C-section (CS) neonates with respect to different time points (in months) was determined by Bray-Curtis diversity index and illustrated through box-whisker plots. The outliers are represented as black dots. The number of samples for each mode of delivery was as follows: VD, *n* = 11 at 1 months, *n* = 10 at 3 months, *n* = 13 at 6 months, and *n* = 9 at 12 months; CS, *n* = 2 at 1 month, *n* = 7 at 3 months, *n* = 12 at 6 months, and *n* = 6 at 12 months.

The interindividual variation of GM between nnVD and nnCS groups, measured employing the Bray-Curtis diversity index (BCDI), was relatively low at T12 (after 1 year) compared to other time points (T1, T3, and T6). Both time (lactation period *viz.* T1, T3, T6, or T12) and mode of delivery (nnVD or nnCS) had a significant impact on nnGM (permutational multivariate analysis of variance [PERMANOVA] *P* value = 0.0002) (Fig. 5). Pairwise analysis of nnCS-NS between two time points revealed that the BCDI was significantly higher between T12 and any other time point during the breastfeeding tenure (i.e., T3 and T6) (false-discovery rate [FDR] *P* < 0.05). However, the BCDI for nnCS-NS was not significantly different for any other pairwise comparison, i.e., when T1 was compared with T3 or T6 or when T3 was compared with T6. In contrast, the BCDI distance for nnVD-NS differed significantly (FDR *P* < 0.05) in all pairwise comparisons (Fig. 5).

**FIG 6:**
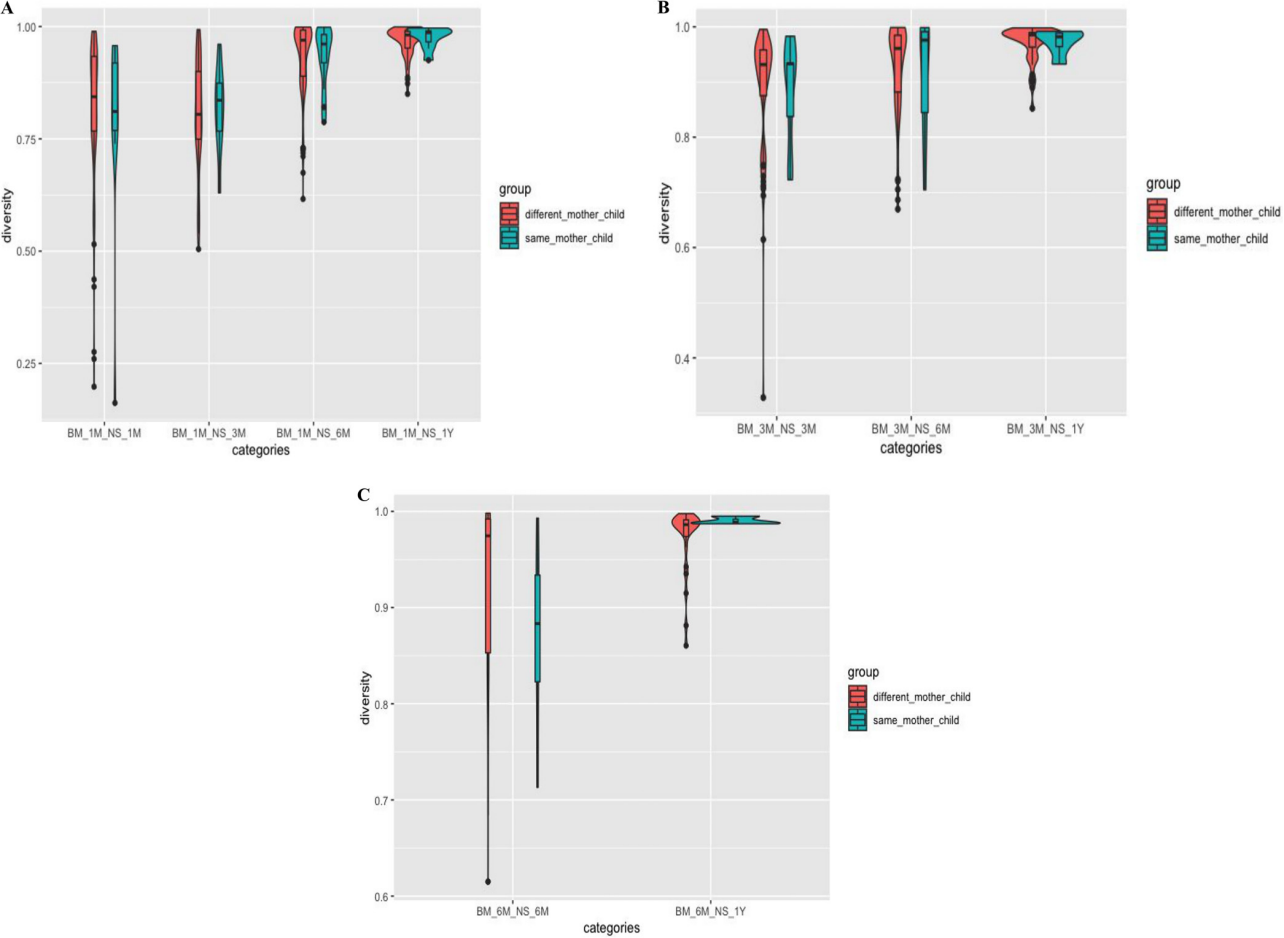
Comparison of the Bray-Curtis dissimilarity index (BCDI) of the microbiome found in NS and BM between the same mother-child pair and between children and different women other than their respective mothers (different mother child). (A) In the first month, BM with different time points of NS, i.e., 1, 3, 6, and 12 months. (B) The 3-month BM with different time points of NS collection, i.e., 3, 6, and 12 months. (C) The 6-month BM with different time points of NS sampling, i.e., 6 and 12 months. The distance between mother-neonate microbiome profiles significantly increased with time, and microbiome diversity of NS at T12 was highest and was quite distinct from BM at any time point. The black dots in the figures represent the outliers.

Exploring the BM and NS association, we compared whether there was any significant difference in microbial diversity between the biological mother-child pair (mother-neonate pair) and between children and different women other than their respective mothers (mother-neonate pseudopair). We did not find any significant difference in the pattern of beta-diversity of these two compared groups (Fig. S5). However, we observed within-group temporal variation, as the BCDI distance between MoVD-BM and nnVD-NS (Fig. 6A to C) within the same time point was less than that compared to between two different time points. The distance between mother-neonate microbiome profiles significantly increased with time, and microbiome diversity of NS at T12 was highest and was highly distinct from BM at any time point (*P* < 0.001). A similar trend was observed for MoVD-BM at T3 that was relatively similar (BCDI small) to that for nnVD-NS at T3 and T6 but significantly different from nnVD-NS at T12, indicating a temporally stable and reduced diversity for 3 months during breastfeeding tenure in respective pairs (BM-NS).

### Breast milk diversity: mode of delivery and lactation period.

**FIG 7:**
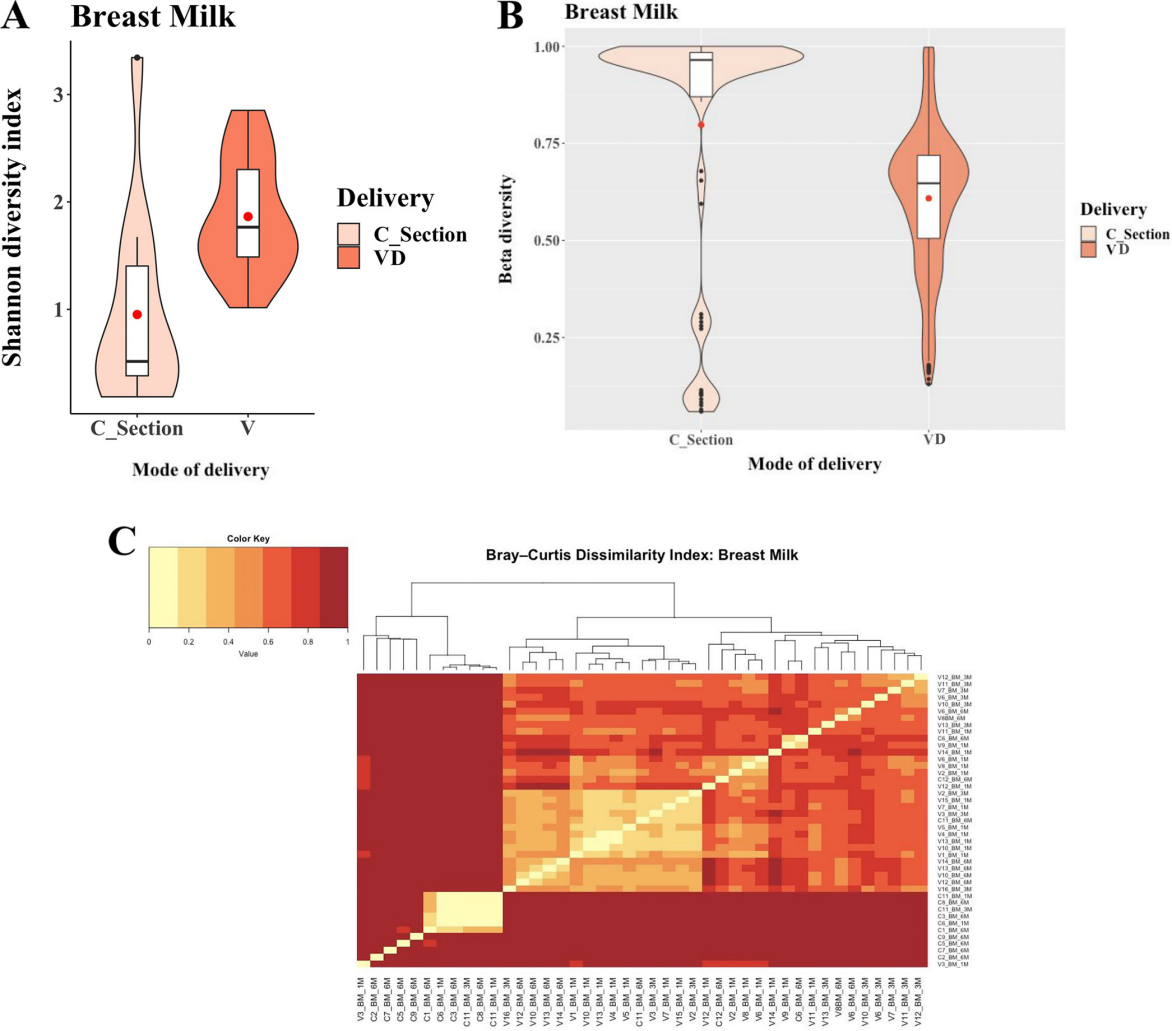
Alpha-diversity of the breast milk among vaginal delivery (VD) and C-section mothers was estimated through a Shannon diversity index (SDI) and illustrated by using violin plots. A Kruskal-Wallis test was employed for statistical comparison. The interindividual variation of the breast milk among VD and C-section mothers was estimated by using the Bray-Curtis diversity index (A) and is depicted by the violin plot (B) as well as a heat map (C).

The SDI of BM among MoVD (SDI, 1.86 ± 0.58; range, 0.63 to 2.85) combined over T1, T3, and T6, i.e., over the entire lactation period, was significantly higher (*P* < 0.003) than that of MoCS (SDI, 0.92 ± 0.88; range, 0.19 to 3.34) (Fig. 7A). Significant differences in SDI were observed between MoVD-BM and MoCS-BM at two time points, T1 and T6 (MoCS-BM_T1, 0.43 ± 0.18, range, 0.3 to 0.56; MoVD-BM_T1, 1.84 ± 0.53, range, 1.01 to 2.85; Kruskal-Wallis test, *P* = 0.02), and T6 (MoCS-BM_T6, 1.06 ± 0.97, range, 0.19 to 3.34; MoVD-BM_T6, 1.94 ± 0.6, range, 1.35 to 2.84; Kruskal-Wallis test, *P* = 0.04), when the neonates were exclusively breastfed (Fig. S6). Overall, the SDI of BM showed a positive longitudinal trend. We observed a higher SDI of BM at T6 than T1, for both MoVD and MoCS, but the differences were found not statistically significant (Kruskal-Wallis test, *P* = 0.8 and 0.4, respectively).

On the other hand, the BCDI of BM revealed higher interindividual variation (*P* = 0.0156) for MoCS-BM (0.8 ± 0.32) compared to MoVD-BM (0.6 ± 0.1) (Fig. 7B). We did not see any temporal monotonic patterns for beta-diversity of BM microbiomes, irrespective of the mode of delivery (Fig. 7C). However, we observed that a few MoCS-BM had a very low BCDI (almost 0), and they had extremely similar microbiome profiles, which were dominated by the OTU corresponding to the genus *Bifidobacterium*.

### Vaginal microbial architecture during onset of labor.

**FIG 8:**
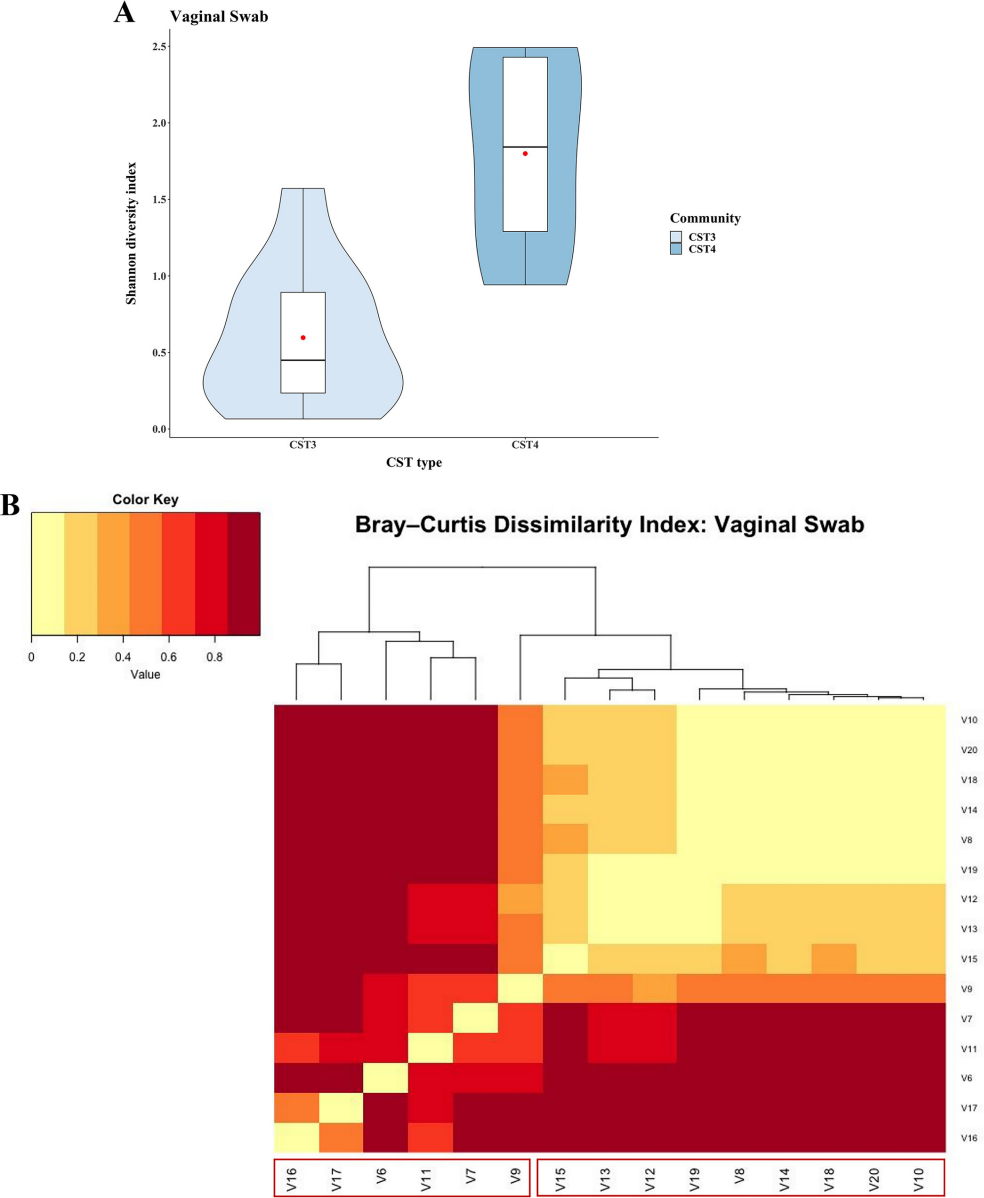
Alpha-diversity of the vaginal microbiota among CST3 and CST4 mothers at the onset of labor (OL) was estimated through the Shannon diversity index (SDI) and illustrated by using violin plots. (A) A Mann-Whitney test was employed for statistical comparison. The red dot represents the mean. (B) A heat map depicting the interindividual variation of the vaginal microbiota among CST3 and CST4 mothers was estimated by using the Bray-Curtis diversity index.

Apart from the BM microbiome, nnVD are exposed to the maternal vaginal canal during birth. Hence, we also investigated the vaginal microbiome, which was collected during the onset of labor (OL). During OL, the SDI of the vaginal microbiome among the mothers (MoVD) ranged from 0.06 to 2.49 (mean, 0.99 ± 0.78). The high percentage of Lactobacillus iners specific OTUs, representative of CSTIII (12), was the most abundant type: 10 of 15 VS samples (range, 45% to 99%). The remaining 5 VS samples were identified as CSTIV, which was defined by a high proportional presence of different microbial communities, such as *Atopium*, *Snethia*, Acinetobacter, and *Gardenella*, and specifically devoid of *Lactobacillus* species (less than 1%). We did not find any VS from CSTI (characterized by Lactobacillus crispatus) or CSTII (characterized by Lactobacillus jensenii). The SDI was higher in CSTIV than in CSTIII (CSTIII, 0.597 ± 0.469; CSTIV, 1.80 ± 0.684; Mann-Whitney test *P* = 0.005) (Fig. 8A). The BCDI of the CSTIII group was low, and VS of mothers belonging to CSTIII were extremely homogeneous at OL, whereas interindividual distances were higher among CSTIV (Fig. 2). We again used MITRE to identify the possible genus, which temporally varied and differentiated between GM of neonates whose mothers belonged to CSTIII versus neonates whose mothers belonged to CSTIV and identified the genus *Megasphaera* as the differentiator.

Further, we asked whether there was any systematic difference in the BCDI between the VM with neonatal GM among the mother-neonate pairs and the pseudopairs. We found there was no significant difference in the pairwise distance between the paired or pseudopaired mother-neonate profiles. However, we did notice that the BCDI distance of neonatal GM with maternal microbiome (BM and VS) was minimal at 1 month after birth, and it significantly increased with time (generalized linear model, *P* < 0.001) (Fig. 8A and B). We found no correlation between the VM and neonatal gut microbiome.

## DISCUSSION

The study mapped the microbial architecture of the neonatal GM and quantified the influence of the maternal microbiome (BM and VS, wherever applicable) as well as the effect of the mode of delivery (CS and VD). We observed that the neonatal GM was altered longitudinally, generally increasing in diversity over time (Fig. 2). The mode of delivery of an offspring was significantly associated with the neonatal GM. Diversity in terms of entropy (SDI) was significantly higher among the VD neonates than among CS babies, at least during the breastfeeding tenure (up to T6); however, the SDI of GM became similar between nnVD-NS and nnCS-NS at T12, possibly because of food diversity and exposure after initiation of solidified food and after the breastfeeding tenure was over (Fig. S3).

The possible reason for higher entropy in nnVD-NS compared to nnCS-NS in early life (breastfeeding tenure) may be because of the interaction with the maternal birth canal. Our inference supports the previous observation from an interventional study which established that inoculation of the VM potentially shaped the neonatal microbiome at early life (9). A similarity of the vaginal microbiome with GM of nnVD has also been documented in multiple studies (13–15). In the present study, the cumulative distribution of *Lactobacillus*, *Prevotella*, or *Sneathia* between VD and CS babies at the age of 1 year showed wider dispersion for CS babies. Similar trends were also observed for Staphylococcus, *Corynebacterium*, and *Propionibacterium* spp. In addition, we also documented the increment of infant age and adaptation of solidified food reduced the difference of GM between nnCS and nnVD. In search of longitudinal factors that contributed to shaping of the neonatal GM, we mapped the BM microbiome at different lactation periods (T1, T3, and T6) after birth. The SDI of the BM microbiota was significantly higher among MoVD compared with the MoCS (nonelective). Higher SDI among MoVD-BM microbiome may be interpreted as a result of labor associated with increased intestinal permeability that is enhanced compared to that for MoCS (16). The postulation for migration of microbial population from gut to BM is supported by interventional studies (17, 18). Studies have documented a probiotic supplement that was introduced to lactating mothers and the supplemented strains observed in maternal GM and BM microflora (19). Another potential reason for reduced alpha-diversity of BM microbiome among MoCSs may be the effect of intrapartum antibiotic prophylaxis, which is are used to reduce risk of maternal wound infection (wound due to C-section). Antibiotic treatment is the usual treatment regimen among MoCS after delivery and it reduces the diversity of the microbial community in maternal intestinal flora (20). Reduced maternal GM also influenced the reduction of bacterial translocation to BM among MoCS. Present observations along with other data postulate that the mode of delivery and subsequent clinical practice (antibiotic administration among nnCS mothers) could alter and reduce its diversity of the NS-GM and MoBM microbial composition.

We also found that the genera *Prevotella*, *Faecalibacterium*, *Bifidobacterium*, Klebsiella and Escherichia were present in all samples irrespective of delivery mode; however, there was a proportional difference observed for certain genera, like *Prevotella*, *Bifidobacterium*, *Megashera*, and Streptococcus. *Bifidobacterium* spp. are well established as a probiotic supplement for its widespread functional potency on vitamin biosynthesis and immune boosting (21). *Prevotella* is crucially involved in hemicellulose catabolism and accelerates pyruvate catabolism (22, 23). The proportion of *Prevotella* was relatively high among nnCS, whereas the abundance for the genus *Megasphaera* was relatively high among nnVD. We found the CST of maternal VM as a critical determinant for the microbial colonization in the neonatal gut flora among the vaginally delivered neonates. The longitudinal change of the proportion of the genus *Megasphaera* has been identified as the differentiating factor between GM of neonates born of CSTIII and CSTIV mothers (Fig. S7). *Megasphaera* possesses unique adaptive features for its survival in the human gut and contributes to various regulatory mechanisms that include bile resistance, stress response systems, and membrane transport through unique sets carbohydrate-active enzymes (CAZymes) (24). *In silico* and *in vitro* analysis showed the potential role of *Megasphaera* on the synthesis of important metabolites, like short-chain fatty acids (butyrate, acetate, formate, and caproate), vitamins, and essential amino acids, which advocates for a potential healthy influence on the host’s gut (24). The same machine-learning approaches revealed that Streptococcus is a crucial OTU that is enriched in the GM of neonates among nnVD (Fig. 4A). Along with the Streptococcus, the genera *Velionella* and *Lactobacillus* coexist during the entire first year of life when breastfeeding is exclusive (Fig S4). It should be noted that Streptococcus, *Velionella*, and *Lactobacillus* are abundantly present in BM also. Coinhabiting bacteria like Streptococcus and *Veillonella* are symbiotically involved in the biosynthesis of lactate, acetic acid, and ethanol for maintaining the acidic pH as an immuno-protectant biofilm in the neonatal gut, which also acts as an innate immune barrier (25).

All pairwise comparisons between MoVD-VM and nnVD-GM revealed that the BCDI was lowest at the first month of birth and monotonically increased with time (Fig. 6A to C). This phenomenon may be because of the enormous influence of VMs in the initiation of neonatal GM; however, the importance of mother’s VM reduces with the increment of age, anatomical maturation, alteration of feeding practice, along with the exposure to other environmental factors. We also saw that the neonatal GM and maternal BM microbiome were dynamic throughout the lactation period (1, 3, and 6 months), and microbial composition was also altered with progress of lactation period. Altered BM human milk oligosaccharide (HMG) components during the lactational period are likely to have a potential role on alteration of BM microbiome diversity, depending on substrate availability of microbes. A reduced dissimilarity index for 3-month VS-GM pairs and BM-GM pairs compared to T6 and T12 may reflect the potential impact of maternal VS and BM microbiome on shaping of neonatal GM at early life. Although it might have long-term ramifications, the dissimilarity increased with the increasing diversity in the neonatal GM as it matured.

We also observed a difference of entropy remained high in breastfeeding tenure (first 6 months of life) among nnVD and nnCS babies. However, while the microbial diversity rapidly increased for individuals after initiation of solidified food, the systematic difference between nnVD-GM and nnCS-GM was reduced. This observation points at the exposure to solidified food significantly contributing to the stability of the neonatal GM in adulthood compared to the lactation period (Fig. S3).

The study postulates that the genera *Bifidobacterium*, *Lactobacillus*, Streptococcus, and *Megasphaera*, which potentially contribute to the neonatal gut, particularly during the lactation period (first 6 months), may be induced through maternal BM and VS. We also did not fail to understand that the influence of breast milk and vaginal microbiome on neonatal GM architecture was not significantly dependent on biological relatedness. The trend of blending relationships between maternal-child microbial populations is more variable with the changing environment, i.e., across the first 1 year. Apart from that, major genera like *Prevotella* and *Faecalibacterium*, which are abundant in all neonatal gut microbiomes after initiation of solidified food but completely absent in maternal source, are likely to be the result of a complex interplay between environment and host food habit, metabolism, and nutrients.

## MATERIALS AND METHODS

### Study participants.

A total of 25 mother-neonate pairs (15 pairs were vaginally delivered and 10 underwent C-section) who experienced term birth were enrolled for the study and followed for 1 year. Our 25 recruited mothers were healthy throughout the sampling period or had not been clinically diagnosed with any kind of infection. None of the mothers had been diagnosed with mastitis or any other kind of infection that could be passed to infants during lactation. Follow-up for neonatal stool sampling was done at <1 month (T1), 3 months (T3), 6 months (T6), and 12 months (T12). BM was collected for 3 time points that included T1, T3, and T6. Vaginal swabs were collected on OL for the mothers who underwent vaginal delivery (*n* = 15). All samples were collected from the same geographical region with matched socioeconomic status from Assam. Mothers predisposed with chronic and infectious lung diseases like tuberculosis, HIV, hepatitis B virus, hypertension, and diabetes, including gestational diabetes and cancer, were excluded from the study. We used sterile cotton swabs for sampling from neonatal stool and vaginal swab (posterior segment) through a trained clinician to avoid interindividual variability on sampling and stored the samples in sterile containers, added a lysis buffer, and shipped samples to the laboratory, maintaining a 4°C temperature. Breast milk was collected in a sterile container (>1 mL) after discarding the first few drops after surface sterilization. DNA samples were isolated strictly within 4 to 6 h of collecting the samples, with the help of a Qiagen Biostic bacteremia kit.

### Ethics statement.

Written informed consent was obtained from all the mothers for them and for their babies as per Institutional Ethics Committee (ICMR-RMRC-NER, the study execution center).

### Sequencing and analysis.

Amplicons were generated through 16s universal primer for variable regions 3 and 4, sequenced on an Illumina-NovaSeq 6000 system (Fig. S8), and analyzed through QIIME (version 2.0) (26). Statistical analysis and graphical representations were done using QIIME, version 2.0, and R, version 4.0.5 (https://www.r-project.org/). Proportions of OTUs were estimated by normalizing OTU-specific read counts with respect to total read counts. The nonparametric Kolmogorov-Smirnov method was used to test the equality of distributions. To compare beta-diversity among different study groups, we employed a PERMANOVA test, considering the data points in beta-diversity were not independent of each other. LDA was performed to discover the bacterial populations that differentiated between two groups. To identify the specific OTUs, which were significantly altered in a temporal fashion, we employed the MITRE method, a supervised machine-learning method.

### Data availability.

The raw data and metadata are available under BioProject accession number PRJNA945262.

## References

[1] Belkaid Y, Hand TW. 2014. Role of the microbiota in immunity and inflammation. Cell 157:121–141. doi:10.1016/j.cell.2014.03.011. 24679531 PMC4056765

[2] Koskella B, Hall LJ, Metcalf CJE. 2017. The microbiome beyond the horizon of ecological and evolutionary theory. Nat Ecol Evol 1:1606–1615. doi:10.1038/s41559-017-0340-2. 29038487

[3] Zheng D, Liwinski T, Elinav E. 2020. Interaction between microbiota and immunity in health and disease. Cell Res 30:492–506. doi:10.1038/s41422-020-0332-7. 32433595 PMC7264227

[4] McKenney EA, Koelle K, Dunn RR, Yoder AD. 2018. The ecosystem services of animal microbiomes. Mol Ecol 27:2164–2172. doi:10.1111/mec.14532. 29427300

[5] Henry LP, Bruijning M, Forsberg SKG, Ayroles JF. 2021. The microbiome extends host evolutionary potential. Nat Commun 12:5141. doi:10.1038/s41467-021-25315-x. 34446709 PMC8390463

[6] Kramer MS, Aboud F, Mironova E, Vanilovich I, Platt RW, Matush L, Igumnov S, Fombonne E, Bogdanovich N, Ducruet T, Collet JP, Chalmers B, Hodnett E, Davidovsky S, Skugarevsky O, Trofimovich O, Kozlova L, Shapiro S. 2008. Breastfeeding and child cognitive development: new evidence from a large randomized trial. Arch Gen Psychiatry 65:578–584. doi:10.1001/archpsyc.65.5.578. 18458209

[7] Stuebe AM. 2015. Does breastfeeding prevent the metabolic syndrome, or does the metabolic syndrome prevent breastfeeding? Semin Perinatol 39:290–295. doi:10.1053/j.semperi.2015.05.008. 26187772 PMC4516665

[8] Jakaitis BM, Denning PW. 2014. Human breast milk and the gastrointestinal innate immune system. Clin Perinatol 41:423–435. doi:10.1016/j.clp.2014.02.011. 24873841 PMC4414019

[9] Mueller NT, Bakacs E, Combellick J, Grigoryan Z, Dominguez-Bello MG. 2015. The infant microbiome development: mom matters. Trends Mol Med 21:109–117. doi:10.1016/j.molmed.2014.12.002. 25578246 PMC4464665

[10] De Seta F, Campisciano G, Zanotta N, Ricci G, Comar M. 2019. The vaginal community state types microbiome-immune network as key factor for bacterial vaginosis and aerobic vaginitis. Front Microbiol 10:2451. doi:10.3389/fmicb.2019.02451. 31736898 PMC6831638

[11] Bogart E, Creswell R, Gerber GK. 2019. MITRE: inferring features from microbiota time-series data linked to host status. Genome Biol 20:186. doi:10.1186/s13059-019-1788-y. 31477162 PMC6721208

[12] Ma ZS, Li L. 2017. Quantifying the human vaginal community state types (CSTs) with the species specificity index. PeerJ 5:e3366. doi:10.7717/peerj.3366. 28674641 PMC5490466

[13] Prince AL, Chu DM, Seferovic MD, Antony KM, Ma J, Aagaard KM. 2015. The perinatal microbiome and pregnancy: moving beyond the vaginal microbiome. Cold Spring Harb Perspect Med 5. doi:10.1101/cshperspect.a023051. PMC4448707 25775922

[14] Kim G, Bae J, Kim MJ, Kwon H, Park G, Kim SJ, Choe YH, Kim J, Park SH, Choe BH, Shin H, Kang B. 2020. Delayed establishment of gut microbiota in infants delivered by cesarean section. Front Microbiol 11:2099. doi:10.3389/fmicb.2020.02099. 33013766 PMC7516058

[15] Dominguez-Bello MG, Costello EK, Contreras M, Magris M, Hidalgo G, Fierer N, Knight R. 2010. Delivery mode shapes the acquisition and structure of the initial microbiota across multiple body habitats in newborns. Proc Natl Acad Sci USA 107:11971–11975. doi:10.1073/pnas.1002601107. 20566857 PMC2900693

[16] Kalbermatter C, Fernandez Trigo N, Christensen S, Ganal-Vonarburg SC. 2021. Maternal microbiota, early life colonization and breast milk drive immune development in the newborn. Front Immunol 12:683022. doi:10.3389/fimmu.2021.683022. 34054875 PMC8158941

[17] van den Elsen LWJ, Garssen J, Burcelin R, Verhasselt V. 2019. Shaping the gut microbiota by breastfeeding: the gateway to allergy prevention? Front Pediatr 7:47. doi:10.3389/fped.2019.00047. 30873394 PMC6400986

[18] Rodríguez JM. 2014. The origin of human milk bacteria: is there a bacterial entero-mammary pathway during late pregnancy and lactation? Adv Nutr 5:779–784. doi:10.3945/an.114.007229. 25398740 PMC4224214

[19] Beck LC, Masi AC, Young GR, Vatanen T, Lamb CA, Smith R, Coxhead J, Butler A, Marsland BJ, Embleton ND, Berrington JE, Stewart CJ. 2022. Strain-specific impacts of probiotics are a significant driver of gut microbiome development in very preterm infants. Nat Microbiol 7:1525–1535. doi:10.1038/s41564-022-01213-w. 36163498 PMC9519454

[20] Yassour M, Vatanen T, Siljander H, Hämäläinen AM, Härkönen T, Ryhänen SJ, Franzosa EA, Vlamakis H, Huttenhower C, Gevers D, Lander ES, Knip M, Xavier RJ, DIABIMMUNE Study Group. 2016. Natural history of the infant gut microbiome and impact of antibiotic treatment on bacterial strain diversity and stability. Sci Transl Med 8:343ra81. doi:10.1126/scitranslmed.aad0917. PMC5032909 27306663

[21] Yoshii K, Hosomi K, Sawane K, Kunisawa J. 2019. Metabolism of dietary and microbial vitamin B family in the regulation of host immunity. Front Nutr 6:48. doi:10.3389/fnut.2019.00048. 31058161 PMC6478888

[22] Franke T, Deppenmeier U. 2018. Physiology and central carbon metabolism of the gut bacterium Prevotella copri. Mol Microbiol 109:528–540. doi:10.1111/mmi.14058. 29995973

[23] de Goffau MC, Jallow AT, Sanyang C, Prentice AM, Meagher N, Price DJ, Revill PA, Parkhill J, Pereira DIA, Wagner J. 2022. Gut microbiomes from Gambian infants reveal the development of a non-industrialized Prevotella-based trophic network. Nat Microbiol 7:132–144. doi:10.1038/s41564-021-01023-6. 34972822 PMC8727306

[24] Shetty SA, Marathe NP, Lanjekar V, Ranade D, Shouche YS. 2013. Comparative genome analysis of Megasphaera sp. reveals niche specialization and its potential role in the human gut. PLoS One 8:e79353. doi:10.1371/journal.pone.0079353. 24260205 PMC3832451

[25] Bäckhed F, Roswall J, Peng Y, Feng Q, Jia H, Kovatcheva-Datchary P, Li Y, Xia Y, Xie H, Zhong H, Khan MT, Zhang J, Li J, Xiao L, Al-Aama J, Zhang D, Lee YS, Kotowska D, Colding C, Tremaroli V, Yin Y, Bergman S, Xu X, Madsen L, Kristiansen K, Dahlgren J, Wang J. 2015. Dynamics and stabilization of the human gut microbiome during the first year of life. Cell Host Microbe 17:690–703. doi:10.1016/j.chom.2015.04.004. 25974306

[26] Caporaso JG, Kuczynski J, Stombaugh J, Bittinger K, Bushman FD, Costello EK, Fierer N, Peña AG, Goodrich JK, Gordon JI, Huttley GA, Kelley ST, Knights D, Koenig JE, Ley RE, Lozupone CA, McDonald D, Muegge BD, Pirrung M, Reeder J, Sevinsky JR, Turnbaugh PJ, Walters WA, Widmann J, Yatsunenko T, Zaneveld J, Knight R. 2010. QIIME allows analysis of high-throughput community sequencing data. Nat Methods 7:335–336. doi:10.1038/nmeth.f.303. 20383131 PMC3156573

