# Supplementary material for: Influence of Maternal Breast Milk and Vaginal Microbiome on Neonatal Gut Microbiome: a Longitudinal Study during the First Year
Source: Microbiol Spectr. 2023 Apr 17;11(3):e04967-22. doi: 10.1128/spectrum.04967-22 (PMC10269640; doi:10.1128/spectrum.04967-22)
Supplement: Supplemental file 1 — Supplemental material. Download spectrum.04967-22-s0001.pdf, PDF file, 1.8 MB [file spectrum.04967-22-s0001.pdf]

Supplementary 1:

Sample ID	Age	Mode of Delivery	Reason of C-Section	Delivery Date	Antibiotics Administered	Sex of Child	Weight of Child	Feeding Status
AB	20	Vaginal delivery	NA	4/3/2017	NA	Male	2.9 KG	Breastmilk
AR	21	Vaginal delivery	NA	15/03/17	NA	Female	2.4 kg	Breastmilk
BD	29	C-Section	Cephalopelvic Disproportion	22/07/16	Ceftriaxone (3 days)	Female	3.63 KG	Breastmilk Only
DM	26	C-Section	Failure to progress in labour	3/6/2016	Ceftriaxone (3 days)	Female	2.84 kg	Breastmilk Only
FB	31	C-Section	Cephalopelvic Disproportion	28/06/16	Ceftriaxone (3 days)	Female	3.08kg	Breastmilk Only
GD	26	C-Section	Cephalopelvic Disproportion	26/04/16	Ceftriaxone (3 days)	Female	4 kg	Breastmilk Only
GR	35	Vaginal delivery	NA	1/3/2017	NA	Female	2.6 KG	Breastmilk
JY	20	Vaginal delivery	NA	9/3/2017	NA	Male	2.8 KG	Breastmilk
NB	21	Vaginal delivery	NA	7/3/2017	NA	Female	3.1 KG	Breastmilk
NL	33	Vaginal delivery	NA	16/03/17	NA	Male	3.0 kg	Breastmilk
NN	20	Vaginal delivery	NA	8/3/2017	NA	Male	2.9 kg	breastmilk
PD	24	C-Section	Failure to progress in labour	24/04/16	Ceftriaxone (3 days)	Female	2.87 kg	Breastmilk Only
PK	23	Vaginal delivery	NA	8/3/2017	NA	Female	2.8 kg	Breastmilk
PP	23	C-Section	Multiple pregnanacy	10/6/2015	Ceftriaxone (3 days)	1 Male,1 Female	2.6kg(m),2.7kg(f)	Breastmilk+ Formula Feed
PR	26	Vaginal delivery	NA	28/02/17	NA	Female	2.9 kg	Breastmilk
RB	21	Vaginal delivery	NA	8/7/2016	NA	Male	1.47 kg	Breastmilk+ Formula Feed
RD	20	C-Section	Failure to progress in labour	25/04/16	Ceftriaxone (3 days)	Female	2.64 kg	Breastmilk Only
RM	23	Vaginal delivery	NA	2/3/2017	NA	Male	2.2 kg	Breastmilk
SB	23	C-Section	Antepartum haemorrhage	8/7/2016	Ceftriaxone (3 days)	Male	1.94 kg	Breastmilk+ Formula Feed
SF	21	C-Section	Antepartum haemorrhage	14/09/16	Ceftriaxone (3 days)	Female	2.4 kg	Breastmilk Only
SJ	31	Vaginal delivery	NA	15/03/17	NA	Female	4.5 kg	Breastmilk
SN	22	Vaginal delivery	NA	16/03/17	NA	Male	2.6 kg	Breastmilk
SR	35	Vaginal delivery	NA	2/3/2017	NA	Male	1.9 KG	Breastmilk+ Formula Feed
ST	28	Vaginal delivery	NA	15/03/15	NA	Female	3.1 kg	Breastmilk
SW	26	C-Section	Multiple pregnanacy	1/10/2016	Ceftriaxone (3 days)	Twin(female)	1.7 kg, 1.74 kg	Breastmilk+ Formula Feed

Supplementary 2:

- Alpha-diversity of neonatal stools (NS) among C-Section and vaginally delivered (VD) infants during their first year of life (combining all four time points) was estimated through Shannon diversity index (SDI) and illustrated by using violin plots. Kruskal-Wallis test was employed for statistical comparison.
- Alpha-diversity is significantly higher among the VD infants as compared to neonates born through C-section.

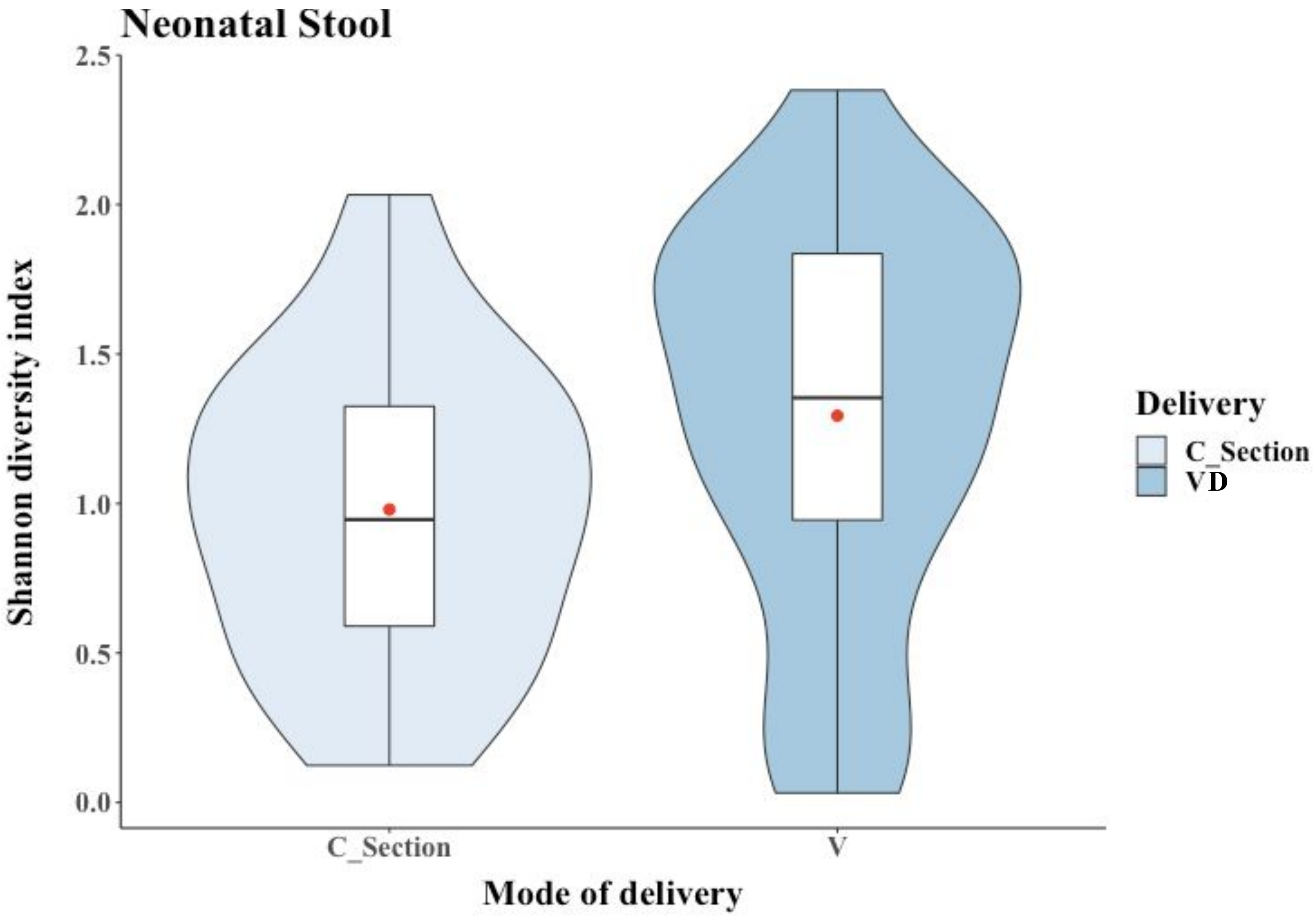

Supplementary 3:

- Alpha-diversity of the neonatal stools among C-Section and vaginally delivered (VD) infants with respect to different time points (months) was estimated through Shannon diversity index (SDI) and illustrated by using violin plots. Kruskal-Wallis test was employed for statistical comparison. The black dots in the figures represent the outliers.
- In the first month after delivery (1M), there is no difference between microbiome of VD and C-section infants
- A significant rise is observed in SDI among VD as compared to CS neonates at the third and six months respectively.
- The difference of GM-SDI becomes reduced among VD and CS at 12th month.

**Neonatal Stool**	**Shannon diversity index**				**Kruskal-Wallis test**
	**C_Section**		**Vaginal**		
	**Mean**	**SD**	**Mean**	**SD**	
**1 Month**	0.64	0.73	0.67	0.57	0.693
**3 Months**	0.82	0.34	1.21	0.46	**0.05096**
**6 Months**	0.8	0.4	1.43	0.52	**0.002775**
**12 Months**	1.64	0.27	1.95	0.3	0.1255
**Kruskal-Wallis test**	**0.006027**		**0.000035**		

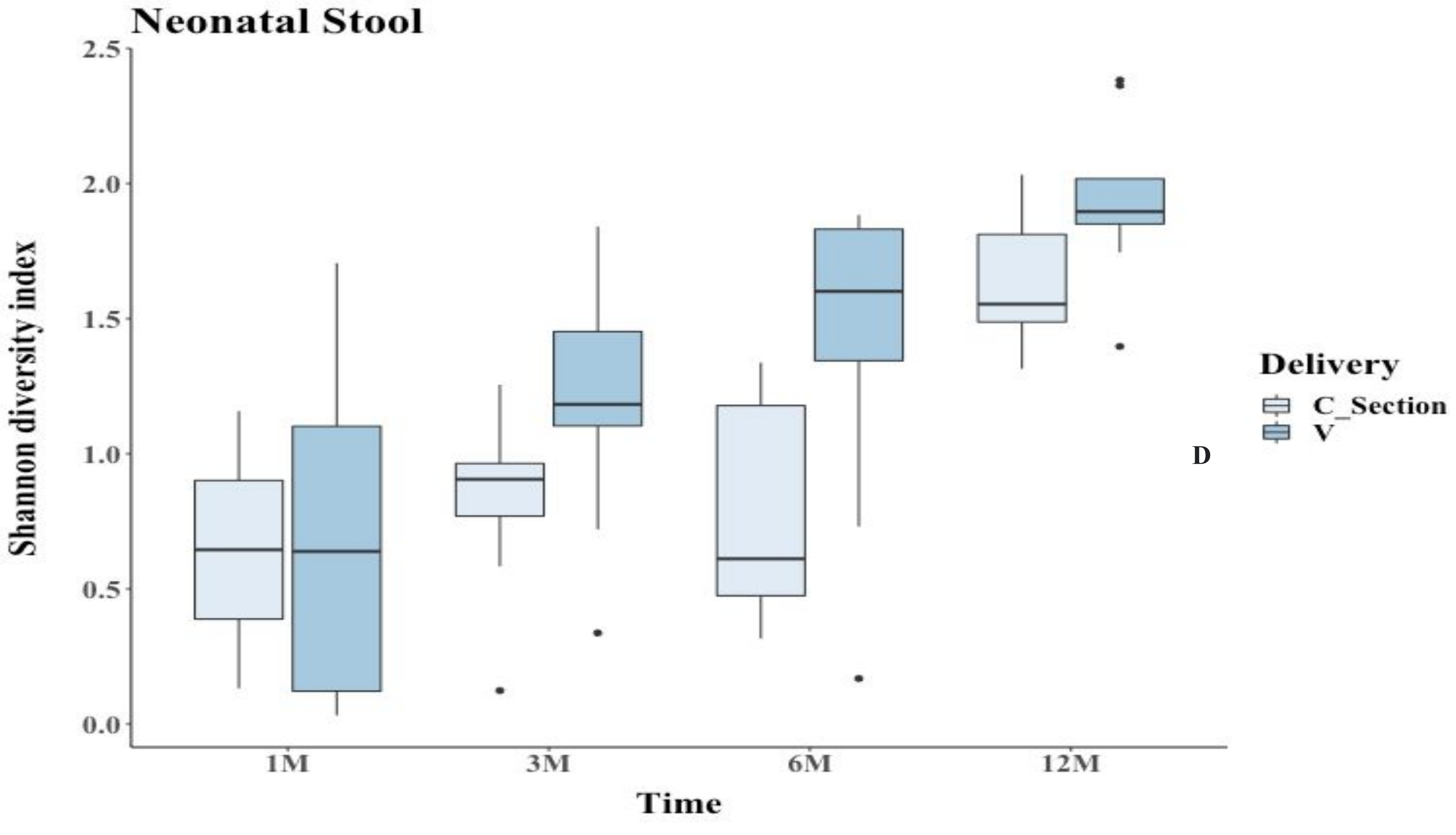

Supplementary 4:

T1-results: We found The study documented that early life GM (within 1 M) is less diverse, primarily majorly dominated by genus Escherechia (>50%-98%) among nVD whereas nCD infant GM is dominated by Bifidobacterium (>10-97%), except for one neonate, for whom and for one case >60% of OTUs of nCD GM remains unassigned. The data reveals that the genus Escherechia () and Streptococcus dominated the BM of among all moVD- BM whereas BM of moCD BM was dominated with the genus Bifidobacterium (range 87.5%-93.5%).

T3-results: Micrococcales (n=1; proportion:>70%), Bifidobacterium (n=2, 88-98%), Sutterella (n=2, proportion: 68.5-79%) and Coprobacillus (n=1, >40%) among the nCD GM at 3 Months of age. In the present study we observed that genus Bifidobacterium dominantly present in a nCD BM (87.5%) and its' twin offspring (87.5% and 93.5%) gut at the age of 3 months. No uniform bacterial pattern is observed for nCD at 3 months of lactation period. In the GM of nVD, the proportion of Escherichia was reduced compared to T1, compared to 1st month compensated by an increaseing the proportion of the bacteria likethat includes Streptococcus, Velionella, Megasphrea and Sutterella among nVD NS. The cumulative proportion of these bacteria was explained >75% of GM among all nVD- NS at 3 months after birth. Following these bacteria in the BM of moVD (n=9) at T3, Wwe observed that the genus Streptococcus (range: 14.7-86%) and Escherichia (range:1.4-9.4 %) to be present in all individuals mVD BM (n=9) . Veillonella iwas present in 70% (7 out of 9) of 3 months' mVD BM with the proportion of ~5%.

T6-results: We observed that Enterobacter, Bifidobacterium, Clostridium, Ruminococcus and Acenetobacter to have a cumulative proportion of explain >80% abundance of all GM among nCD at T6. 6 months of age; We observe a higher interindividual variation persists. The data reveals t The genus Bifidobacterium was present in 50% of samples and we find 3 mother-offspring pairs, where the offspring's gut is individuals' Gut, dominated by the genus Bifidobacterium (proportion>50%) and where the proportion of Bifidobacterium was also remains high among their mothers (range:60.7-90.5%). An OTU from Enterobacter, is present in 8 (80%) individuals, was observed in high frequency and the same dominated in among 4 individuals (38.4-88.2%). Clostridium and Acenetobacter were the most frequent bacteria dominated in the GM of nCD GM observed for one individual each. Ruminococcus was present in 4 individuals (proportion: 0.003-26.6%). In the interval of T1 to T3, Tthe proportion of the genus Bifidobacterium increased in elevated among GM of all nVD samples. of nVD- GM compared to 1 months and 3 months' nVD GM and The Bifidobacterium co-exists with Escherechecia, Bacteroidetes, Suterella and Velionella. The observed proportion of Bifidobacterium remains <5% among 4 individuals and rest 9 samples acuuried >10% of proportion in the nVD GM. One individual's GM is dominated by Eschechechia around 97% and for the remaining 14 samples the proportion remains <50%. The proportion of Veillonella ranges 10-27% among 7 individuals(~50% of samples) and for other 6 individuals, it remains within 2-5% of GM. Bacteroidetes dominated among 4 samples(~30%) with the proportion of >40%.

The genus Escherechia, Streptococcus, Bacteroidetes, Bifidobacterium and Velionella explains >75% of GM of all 13 individuals at the age of 6 month among nVD. mVD BM majorly dominated by Streptococcus and Velionella and the cumulative proportion ranges from 23.6-74.8%.

The genus Lactobacillus, Bifidobacterium and Escherechia present in all mVD BM with the proportion of <1%. The GM at the age of 1 year majorly dominated by the genus Prevotella, Escherechia, Lactobacillus and Faecalibacterium with its cumulative abundance >50%. The genus Prevotella, Faecalibacterium and Escherichia are present in all samples irrespective of delivery mode. The proportion of Prevotella is relatively high among nCD (5 out of 12 samples the proportion of the same is >50%) compared to nVD(2 out of 9 samples the proportion of the same is >50%). The proportion of the genus Escherichia (proportion 1-47% among nCD and 1-50% among nVD) and Faecalibacterium (highest proportion >15% among nCD and 21% among nVD) remains same among nVD and nCD. Apart from that, the genus Megaspharea, Bacteroidetes and OTUs from Enterobacteriaceae are present in the majority of samples with the proportion >1% - <25%. The proportion of Megashera is higher among nVD (highest proportion >25%) compared to nCD (highest proportion <5%). The genus Klebsiella and Bifidobacterium present in all samples (nVD and cVD) with the proportion <1~5%. (Supplemental Figure 3.1-3.3)

Supplemental Figure 4.1:
List of OTUs that are present in neonatal stool across 1 year. Also marking their presence in breast milk and birth canal.

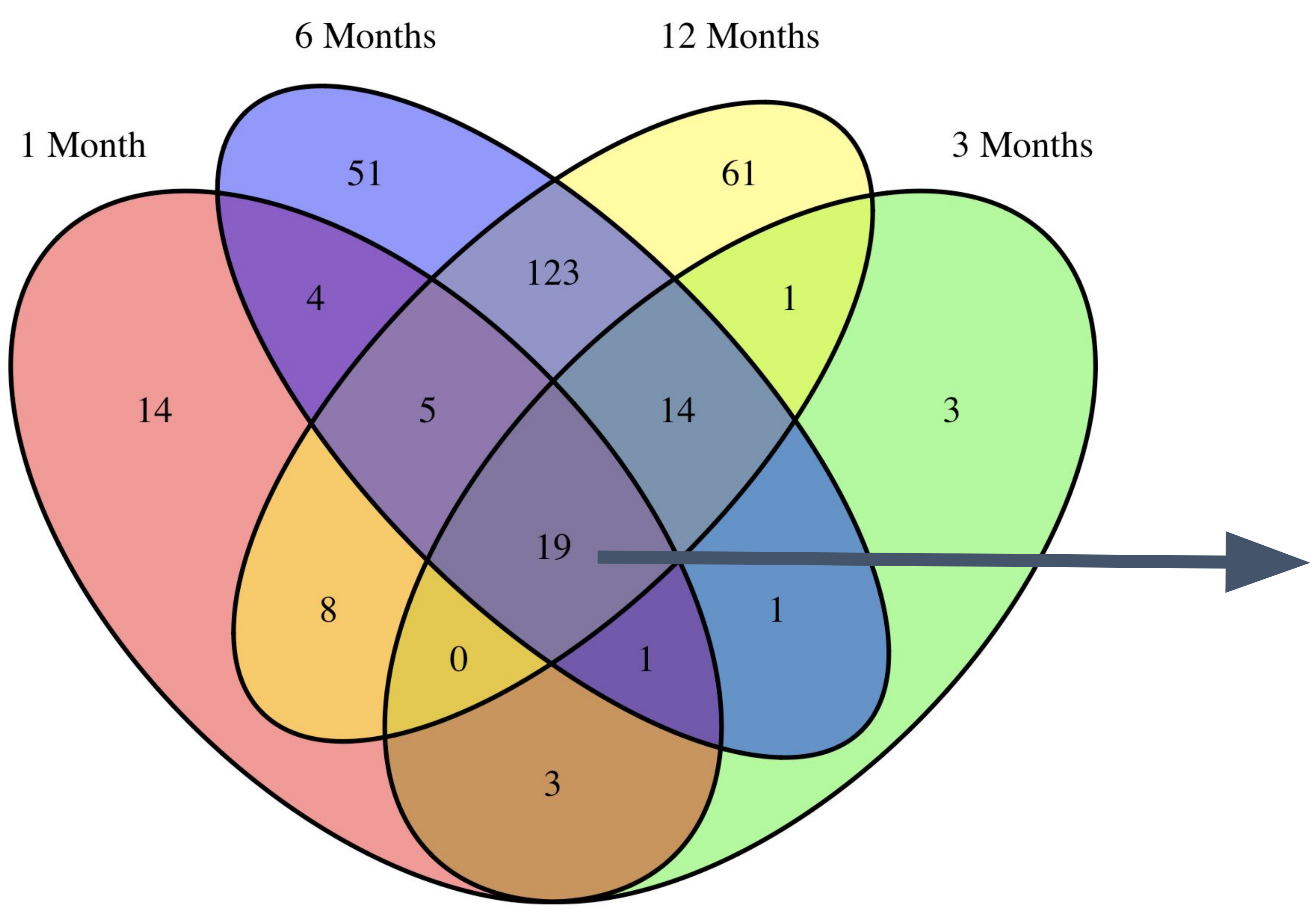

Present in		
NS	BM	VS
Staphylococcus	✓	✓
Enterococcus	✓	✓
Lactobacillus	✓	✓
Streptococcus	✓	✓
Dialister	✓	✓
Megasphaera	✓	✓
Veillonella	✓	✓
Enterobacteriaceae	✓	✓
Klebsiella	✓	✓
Acinetobacter	✓	✓
Pseudomonas	✓	✓
Gemellaceae	✓	-
Granulicatella	✓	-
Erysipelotrichaceae	✓	-
Eubacterium	✓	-
Sutterella	✓	-
Enterobacteriaceae.Other	✓	-
Haemophilus	✓	-
Catenibacterium	-	-

Supplemental Figure 4.2:

Alteration of correlation among OTUs of neonatal gut microbiome compared between exclusively breast feed tenure (1-6 Months) and after initiation of solidified food (12 Months).

- Negative correlation observed for Veillonella and Streptococcus with Bifidobacterium during breast feed tenure that shifted to positive correlation after initiation of solidified food (12 months).
- Shift of positive to negative correlation documented for the bacterial pair of Prevotella-Bifidobacterium and Klebsiella-Lactobacillus between exclusively breast feed tenure (1-6 Months) and after initiation of solidified food(12 Months).

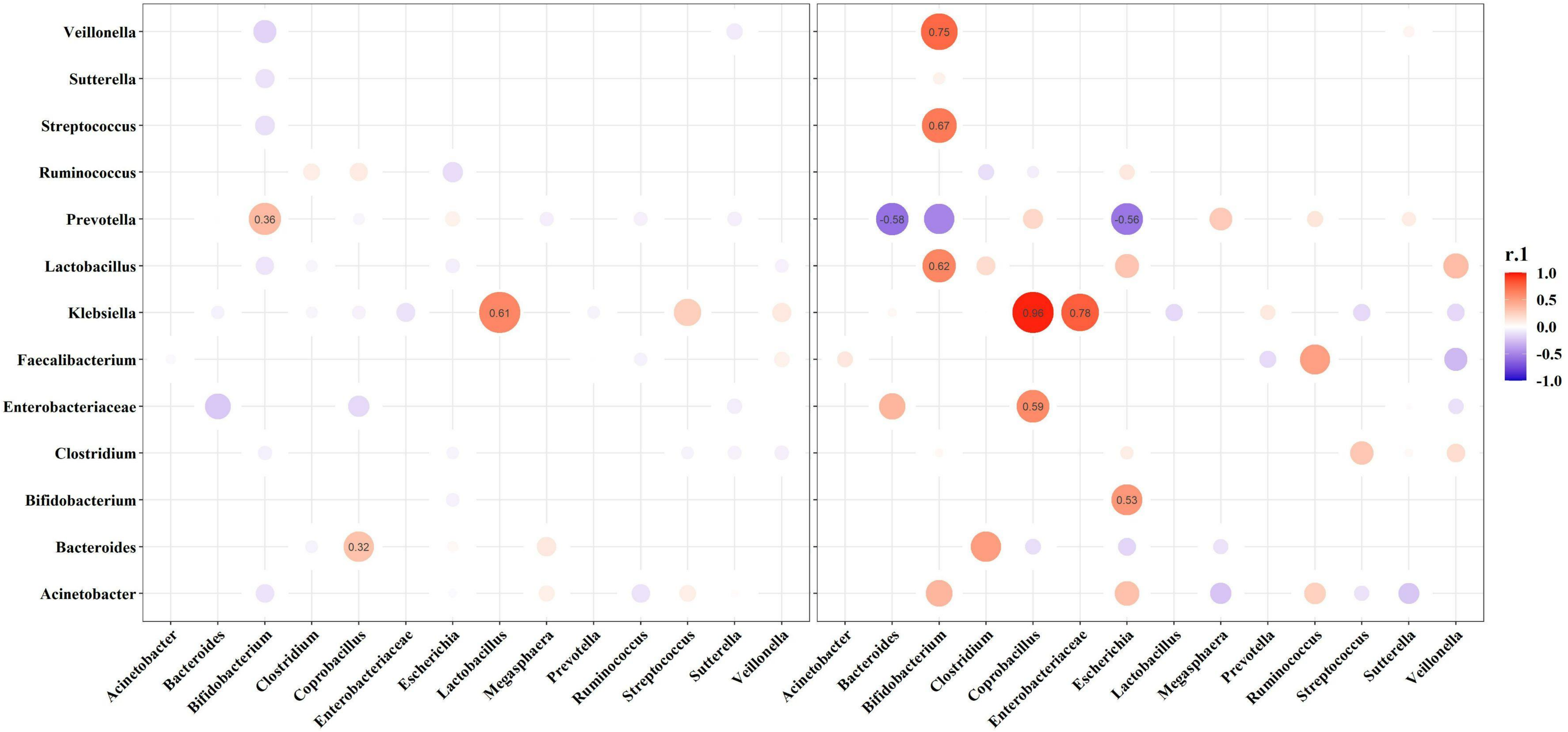

Supplemental Figure 4.3:

Heatmap of OTUs that present throughout the 1 year of life after birth at neonatal gut.

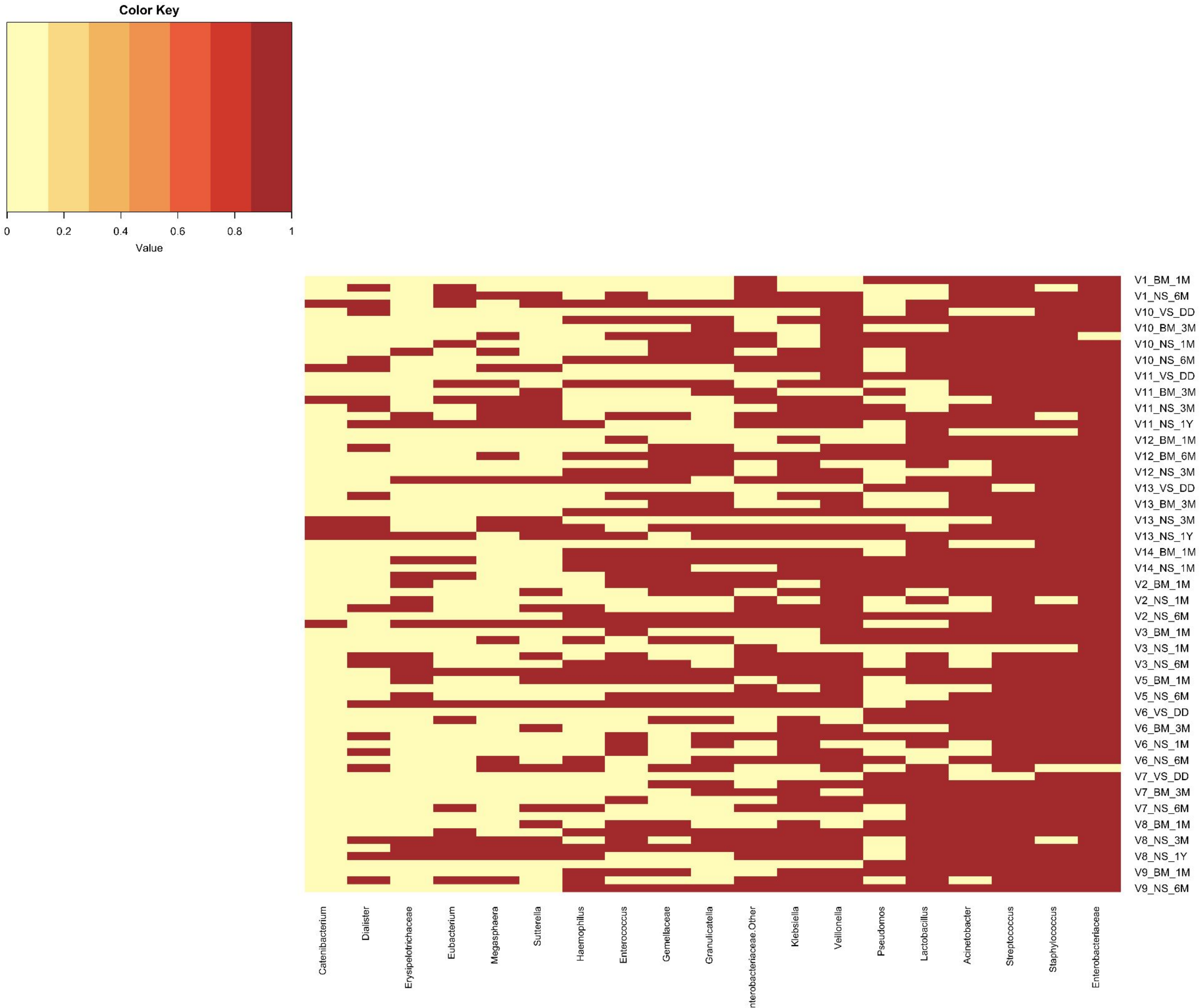

Supplemental Figure 4.4:
Proportion of key bacteria in neonatal stool in different time point.

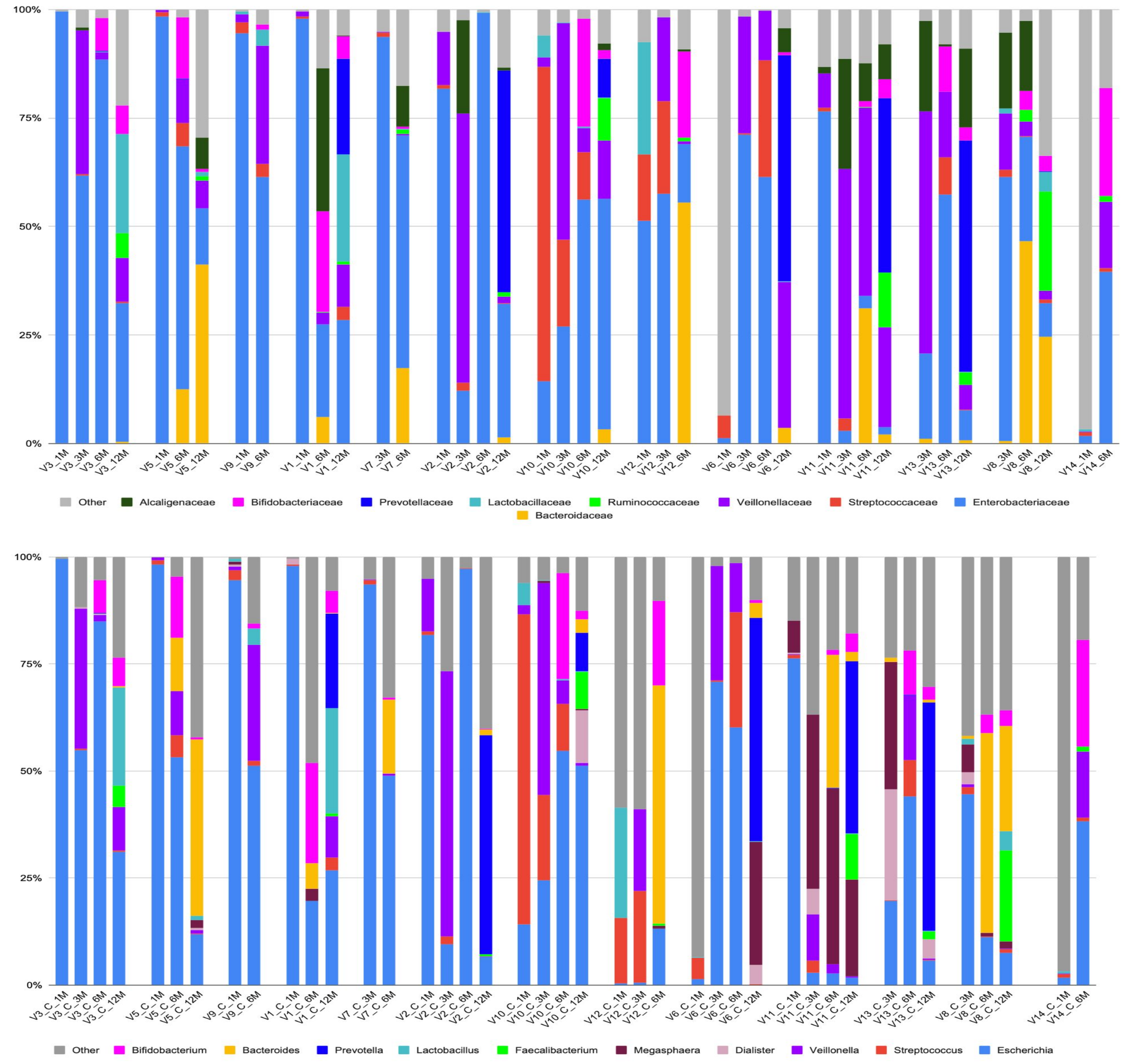

Supplementary 5:

- Bray-Curtis dissimilarity index (BCDI) between the same mother-child pair (mother-neonate pair) and between children and different women other than their respective mothers (mother-neonate pseudo-pair).
- BCDI less among same mother-child pair compare to pseudo pair.
- BCDI increase with time.

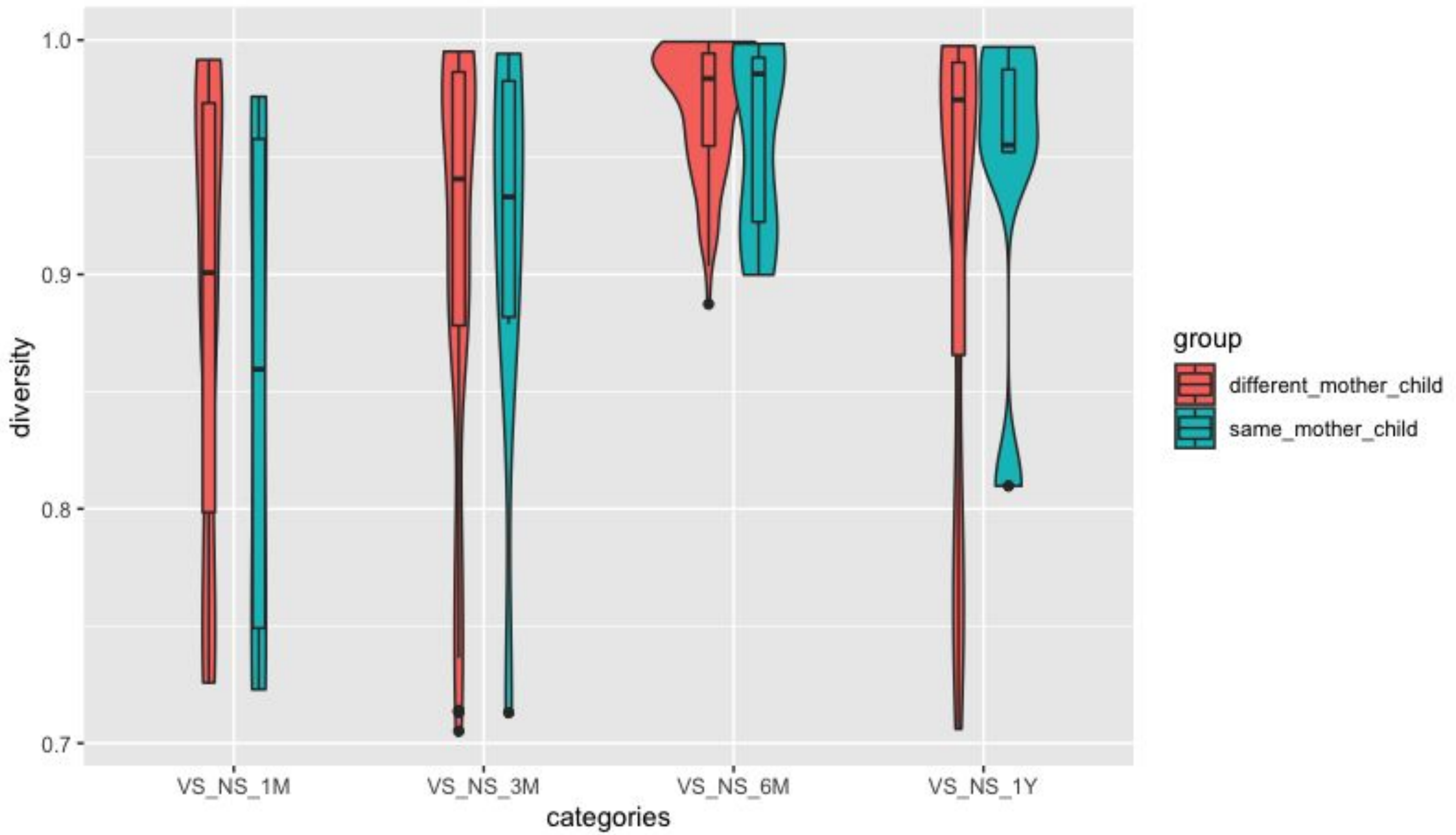

Supplementary 6: Significant difference of SDI between MoVD-BM and MoCS-BM at two time points 1M(<1 month after birth) and 6M(~6 months after birth).

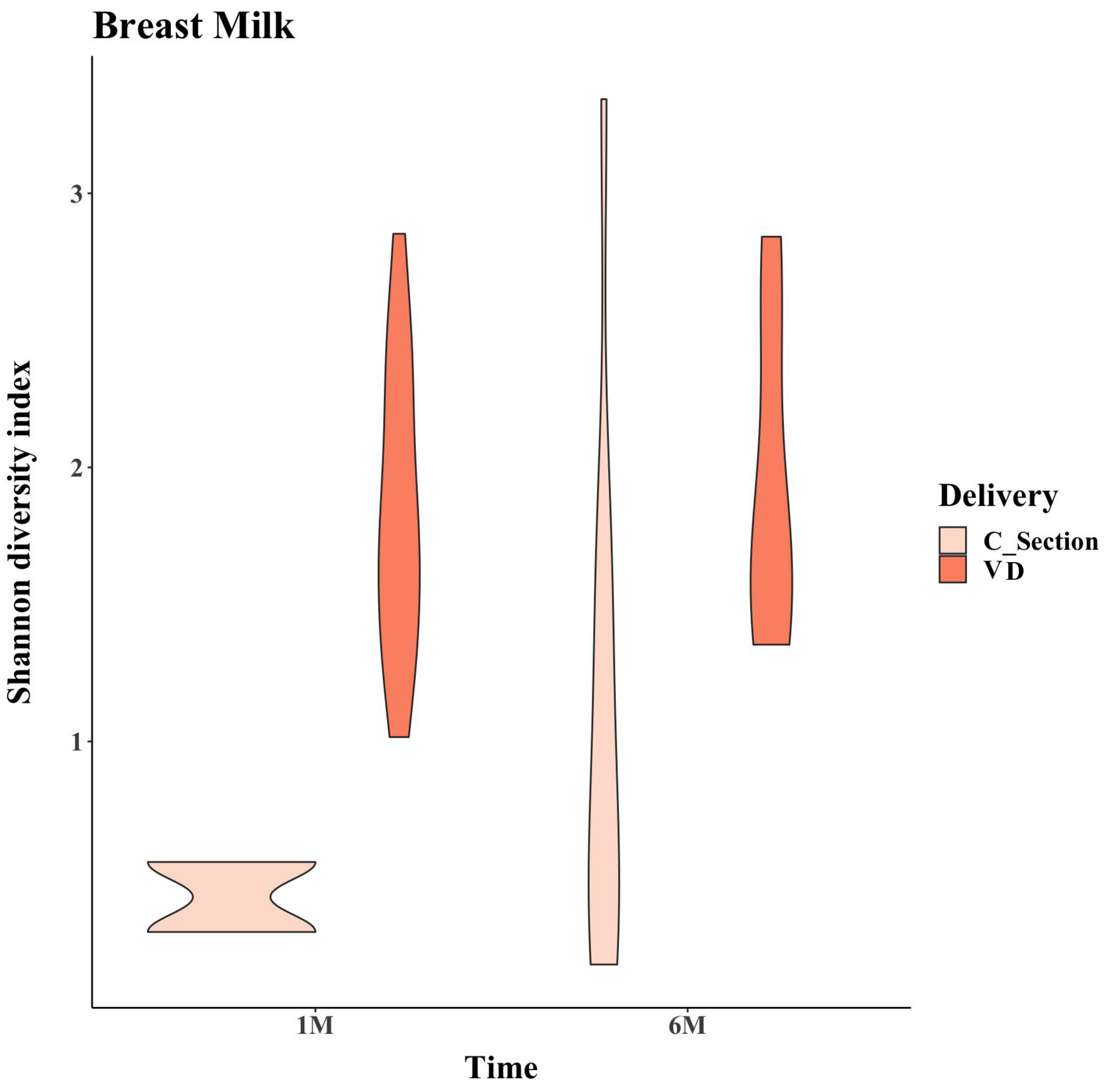

Supplementary 7:

- Time series analysis of longitudinal data on neonatal GM through MITRE based machine learning approaches revealed that the genus differentiated the CST III and CST IV derived nVD GM
- The CSTIV VM community influence the colonization of the genus Megasphaera.
- The black dashed line represents an average abundance of 0.0508 which classifies the CST III and IV groups.

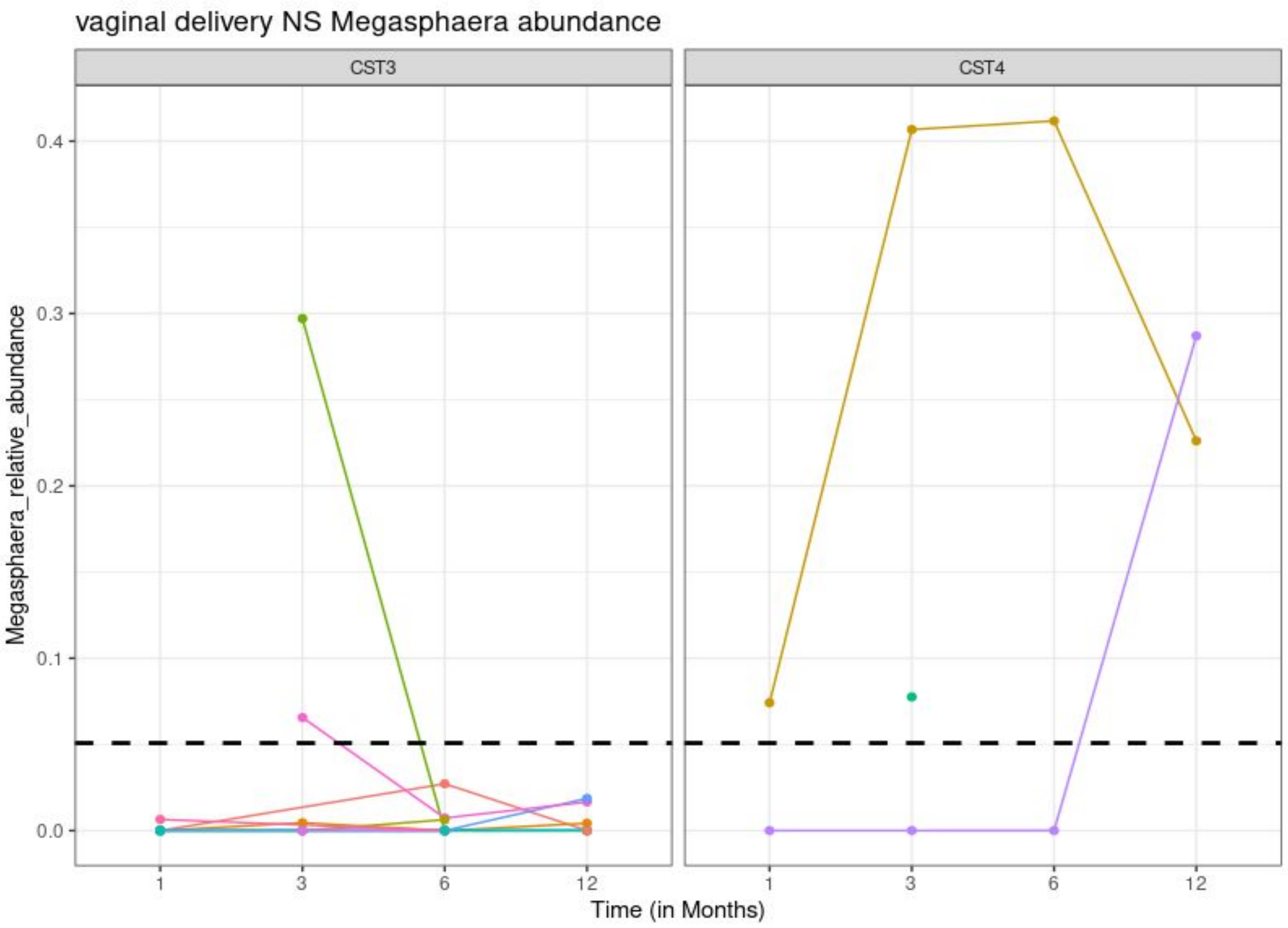

Supplementary 8:

Methodology for 16S Amplicon Sequencing (V3V4)

Amplicon Primers:

- The gene-specific sequences used in this protocol target the 16S V3 and V4 region. They are selected from the Klindworth et al. publication (Klindworth A, Pruesse E, Schweer T, Peplles J, Quast C, et al. (2013) Evaluation of general 16S ribosomal RNA gene PCR primers for classical and next‐generation sequencing‐based diversity studies. Nucleic Acids Res 41(1).) as the most promising bacterial primer pair. Illumina adapter overhang nucleotide sequences are added to the gene‐specific sequences. The full length primer sequences, using standard IUPAC nucleotide nomenclature, to follow the protocol targeting this region are:

16S Amplicon PCR Forward Primer = 5'
TCGTCGGCAGCGTCAGATGTGTATAAGAGACAGCCTACGGGNGGCWGCAG
16S Amplicon PCR Reverse Primer = 5'
GTCTCGTGGGCTCGGAGATGTGTATAAGAGACAGGACTACHVGGGTATCTAATCC

- This method can also be utilized to target other regions on the genome (either for 16S with other sets of primer pairs, or non‐16S regions throughout the genome; ie any amplicon). The overhang adapter sequence must be added to the locus‐specific primer for the region to be targeted (Figure 1). The Illumina overhang adapter sequences to be
- added to locus‐specific sequences are:

Forward overhang: 5’ TCGTCGGCAGCGTCAGATGTGTATAAGAGACAG‐[locus specific sequence]
Reverse overhang: 5’ GTCTCGTGGGCTCGGAGATGTGTATAAGAGACAG‐[locus specific sequence]

Sequencing details:

16srRNA Gene Sequencing Library Preparation and Sequencing Run:

Genomic DNA was used as the input material for 16s rRNA gene sequencing. Fusion primer was used to amplify V3-V4 region and followed by index PCR using Nextera XT index kit (illumina). Quality of final libraries was checked using high sensitivity D1000 screen tape in 2200 TapeStation (Agilent) and final library quantification was performed in Qubit Fluorometer. Paired end (2 x 250 bp) sequencing of these libraries were performed in Novaseq 6000 (Illumina) SP Flowcell.
